# miR‐185‐5p Derived From hUC‐MSC Exosomes via Suspension Culture Under Hypoxic Conditions Promotes Scarless Wound Healing in Mice by Precisely Regulating Collagen I/III Regeneration

**DOI:** 10.1002/advs.202516120

**Published:** 2026-02-19

**Authors:** Lihua Yang, Yiqun Sun, Qinbiao Yan, Ali Mohsin, Kamran Ashraf, Senyi Gong, Weifeng Li, Touseef Ur Rehman, Yuwen Hu, Jinzhao He, Yu Liu, Meijun Ding, Lin Qi, Meijin Guo, Ke Xue

**Affiliations:** ^1^ State Key Laboratory of Bioreactor Engineering East China University of Science and Technology Shanghai Shanghai China; ^2^ Department of Burn and Plastic Surgery Hainan Western Central Hospital Danzhou Hainan China; ^3^ Theseus Biotechnology (Shanghai) Co., Ltd Shanghai Shanghai China; ^4^ Department of Burn and Plastic Surgery Affiliated Hospital of West Anhui Health Vocational College Luan Anhui China; ^5^ Department of Radiology Huadong Hospital Fudan University Shanghai Shanghai China; ^6^ Department of Plastic and Reconstructive Surgery Shanghai Ninth People's Hospital Shanghai Jiao Tong University School of Medicine Shanghai Shanghai China

**Keywords:** collagen ratio, hypoxic exosomes, miR‐185‐5p, scarless healing, three‐dimensional suspension culture

## Abstract

Hypertrophic scars have long posed a significant challenge in wound healing, primarily because of an imbalance between Type I (COL‐I) and Type III (COL‐III) collagen caused by excessive fibroblast activation. Existing treatments cannot directly regulate collagen formation to intervene in scar hyperplasia. This study is the first to elucidate the key mechanism through which human umbilical cord‐derived mesenchymal stem cell (hUC‐MSCs) exosomes expressing miR‐185‐5p achieve scarless healing through remodeling of the collagen‐type ratio. In this method, a hypoxic three‐dimensional (3D‐HO) suspension culture system that more closely mimics the in vivo microenvironment was established, and functional exosomes with enhanced physiological activity and higher yields were harvested. In vivo experiments demonstrated that 3D‐HO exosomes significantly improved the COL‐III/COL‐I regeneration ratio during mouse wound healing. Mechanistic studies revealed that the RhoA/YAP signaling axis plays a key regulatory role in the collagen regeneration ratio. Further molecular analysis revealed enrichment of miR‐185‐5p in 3D‐HO exosomes, which directly target RhoA to regulate fibroblasts. Both in vivo and in vitro functional interventions confirmed miR‐185‐5p in maintaining collagen type balance. In summary, this study demonstrates that microenvironment‐enhanced hUC‐MSCs exosomes remodel the composition of different types of collagen via the miR‐185‐5p–RhoA/YAP signaling axis, thereby driving scarless healing.

## Graphical abstract

miR‐185‐5p derived from UC‐MSC exosomes via hypoxic suspension culture promotes scarless wound healing in mice by precisely regulating collagen I/III regeneration. Exosomes derived from UC‐MSCs under a three‐dimensional bioreactor and hypoxic conditions were injected into the skin wounds of mice. B miR‐185‐5p in 3H‐EXOs can decrease the production of RhoA and the expression of YAP while inhibiting the overexpression of COL‐I and thereby increasing the production of COL‐III to promote scarless wound healing.

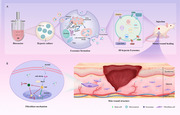

## 1 Introduction

Hyperplastic scarring is a pathological skin condition that develops following wound healing. Most patients with hyperplastic scars present with pronounced tissue growth, redness, firmness, and painful itching at the wound site, which significantly impair their work and social lives [1, 2]. Hyperplastic scars form through a complex mechanism, and their treatment remains challenging. The use of intrascar injections, fractional laser therapy, and surgical excision combined with radiotherapy can provide only temporary relief from symptoms [3, 4]. Furthermore, angiogenesis and re‐epithelialization are the main approaches for promoting wound healing. The formation of new blood vessels is essential for the delivery of oxygen and nutrients to the wound site to supply energy to epithelial cells at the wound margins, enabling their migration through the wound to form a continuous layer for re‐epithelialization [5, 6]. However, while these methods can facilitate rapid wound healing, they do not directly regulate or inhibit scar formation. Compared with that in normal skin extracellular matrix (ECM), the ratio of collagen I (COL‐I) and collagen III (COL‐III) in wound tissue is disrupted. Specifically, the overexpression of COL‐I has been identified as a key factor in ECM fibrosis and scarring [7, 8, 9]. Consequently, keloid formation can be prevented and inhibited at the source by altering the COL‐I to COL‐III ratio during regeneration, specifically by increasing the COL‐III content and reducing the COL‐I content during wound healing.

Additionally, fibroblasts play a critical regulatory role in determining the proportion of collagen [10]. Among them, excessive activation of myofibroblasts by fibroblasts will aggravate the formation of scars on the wound surface [11]. Studies have found that mechanical stimuli such as increased wound tension and matrix hardening can drive the upregulation of *α*‐SMA through mechanical transduction pathways such as RhoA/ROCK, promoting its differentiation into myofibroblasts and continuous contraction [12]. In addition, TGF‐*β*1, PDGF, CTGF and persistent inflammation‐related factors (such as IL‐6/IL‐1*β*/TNF‐*α*, M2 macrophage signaling) will further amplify pro‐fibrotic transcriptional programs and ECM deposition [13]. However, the inflammatory response plays multiple important roles throughout the entire process of wound healing and cannot be directly intervened in or regulated, which may lead to further disorders in wound healing. Therefore, it is expected that the excessive activation of fibroblasts can be regulated by reducing mechanical signal responses, thereby achieving ECM reconstruction of fibroblasts.

Exosomes are excellent natural biological delivery materials that are defined as small extracellular vesicles with diameters ranging from 30 to 150 nm. They usually originate from various cell types. Among them, human umbilical cord mesenchymal stem cells (hUC‐MSCs) are regarded as an ideal source for obtaining high‐quality exosomes because of their strong paracrine activity and immunomodulatory ability. Exosomes carry a variety of active proteins and microRNAs [14, 15, 16]. Among them, these miRNAs are the secret weapons that enable exosomes to have multiple repair functions. Exosomes are generated by the endosomal sorting complex required for transport (ESCRT) pathway, and their unique phospholipid bilayer structure endows them with high membrane fusion efficiency, facilitating intercellular signal transduction and cargo delivery [17, 18]. In addition to their specific molecular structure, the functions of exosomes vary depending on the cell culture method. The cell growth microenvironment (ECM) plays a pivotal role in influencing cell behavior. Cells within the ECM are affected by factors such as ECM stiffness, viscoelasticity, and pore size, and they experience repetitive push and pull forces during adhesion and migration. This mechanical force feedback modulates intracellular signaling through changes in the expression of cell surface receptors (such as integrins), affecting transcription and phenotypic expression [19, 20, 21, 22]. This reciprocal interaction, known as cell–ECM mechanotransduction, is a key process in three‐dimensional (3D) cultures. Compared with two‐dimensional (2D) planar culture, 3D matrix culture not only increases the growth area available to cells but also alters the physiological and functional state through mechanical signaling in the ECM [23, 24, 25]. Although 3D culture systems enable stem cells and their exosomes to secrete higher levels of regenerative factors, thereby enhancing tissue repair, cartilage regeneration, and immunomodulation [26, 27], whether such approaches can precisely remodel the ECM composition remains unclear. In particular, few studies have investigated whether exosomal miRNAs can directly regulate collagen subtype conversion or elucidated the underlying mechanisms governing the COL‐III/COL‐I balance.

Moreover, hypoxic culture is used to stimulate cell proliferation and differentiation under oxygen concentrations of 1%–5%. Under such conditions, cells adapt their respiratory metabolism by enhancing the activity of glycolytic metabolic pathways to compensate for the effects of low oxygen levels on their physiological functions [28, 29]. The hypoxia‐inducible factor Hif‐1*α* has been shown to improve outcomes in cardiovascular disease, neurological disease, and wound healing by activating and promoting the expression of vascular endothelial growth factor (VEGF)‐related signaling pathways. This stimulation leads to endothelial cell proliferation and migration and the formation of new blood vessels, thereby facilitating angiogenesis [30, 31, 32, 33]. It has been demonstrated that MSC‐derived exosomes under hypoxic conditions have beneficial effects on wound vascular regeneration and macrophage polarization [34]. However, a single treatment with hypoxic exosomes has a limited effect on wound healing, and the effects of hypoxic exosomes on collagen regeneration remain unclear. Therefore, exploring whether the collagen ratio and collagen regeneration during wound healing can be modulated by culturing cells under hypoxic conditions in a 3D system is important.

**FIGURE 1:**
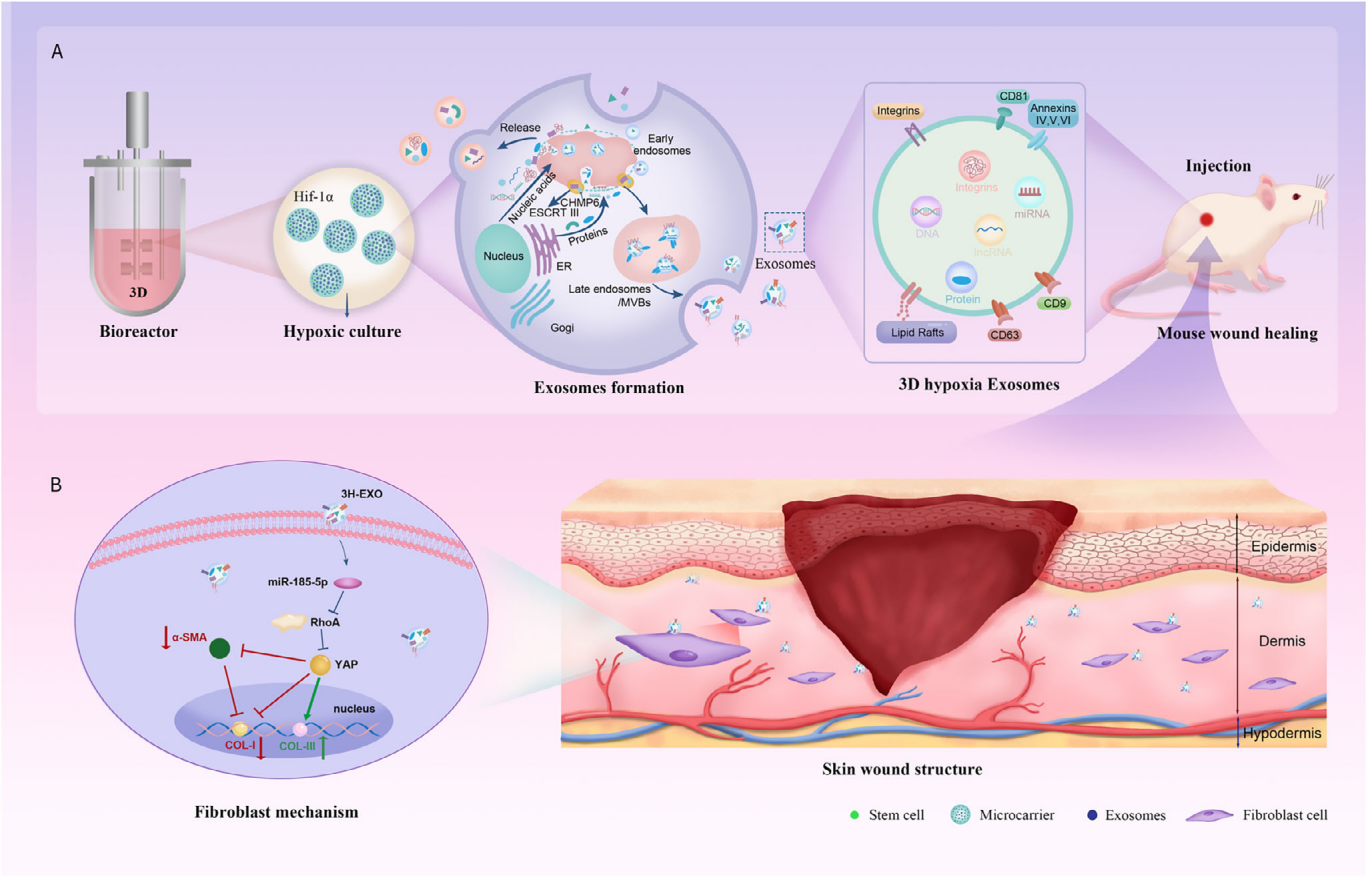
miR‐185‐5p originating from UC‐MSC‐derived exosomes via hypoxic suspension culture promotes scarless wound healing in mice by precisely regulating collagen I/III regeneration. Exosomes derived from UC‐MSCs under a three‐dimensional bioreactor and hypoxic conditions were injected into the skin wounds of mice. B miR‐185‐5p in 3H‐EXOs can decrease the production of RhoA and the expression of YAP while inhibiting the overexpression of COL‐I and thereby increasing the production of COL‐III to promote scarless wound healing.

On the basis of the above reasoning, this study utilized 3D hypoxic culture to prepare mesenchymal stem cell‐derived exosomes (3H‐EXO) with precise ECM regulatory capacity and systematically evaluated their potential for scarless wound healing in mouse wound models. Through miRNA sequencing, functional intervention, and mechanistic validation, we demonstrated that 3H‐EXOs regulate collagen type switching via miR‐185‐5p, thereby reconstructing the ECM structure and promoting scarless healing. This research provides a novel strategy for achieving precise ECM modulation through exosome engineering in skin regeneration (Figure 1a,b).

## 2 Experimental Section

### 2.1 Cell Culture

Human umbilical cord mesenchymal stem cells (hUC‐MSCs) were obtained from Passage 3 (P3) cells maintained in our laboratory. All experiments were approved by the Ethics Committee of the National Academy of Science and conducted in accordance with the guidelines established by the Ministry of Health of the People's Republic of China. The oxygen content in a standard carbon dioxide incubator is typically 21%, and an oxygen percentage ≤ 5% is considered hypoxic. Furthermore, continuous hypoxic culture and cycle stimulation have been shown to elicit stimulatory effects on cell growth and metabolic functions, including the secretion of vesicles (exosomes) with different functions. In this study, exosomes were isolated from cells cultured under continuous hypoxic conditions. Notably, healthy, proliferating cells are needed for the procurement of these vesicles.

The MSCs were centrifuged for 5 min at a ratio of 1:30 in phosphate‐buffered saline (PBS) at 500 × *g*, and the pellet was resuspended in *α*‐MEM (Suzhou Youyi Biotech, China) supplemented with + 5% serum‐free culture medium (AventaCell BioMedical, USA) to achieve a final concentration of 8 – 10 × 10^4^ cells/mL (CountStar, Alit Biotech, China). T‐175 flasks were used for 2D culture, and normoxic culture was carried out in a 5.0% CO_2_ incubator (3111; Thermo, US) at 37°C for 72 to 80 h. The 2D hypoxia group (2D‐HO) was maintained in a triple‐gas incubator (Cellx 170MG, RADOBIO, China) under a 5.0% O_2_ atmosphere. For 3D cultivation, a 1.0 L bioreactor equipped with a marine impeller was used for hUC‐MSC growth. The dissolved oxygen (DO) tension was automatically controlled within a range from 40% to 60%. In the normoxic group, the DO tension was maintained at 60% ± 5%; in the hypoxic group, the DO tension was maintained at 40% ± 5%. The culture pH was maintained at 7.2 ± 0.2 throughout the entire process. The duration of cultivation was approximately 96 h ± 5 h. Microcarriers (Corning, USA) with a diameter of 250 µm at a concentration of 1.0 g/L were added at the beginning of incubation at a density of 5 – 8 × 10^4^ cells/mL. The culture was initially agitated at 35 rotations per minute (rpm) for 5 min, followed by 55 min at 0 rpm, for a total cycle of 7 h at the start of incubation. A constant rotation speed of 35 rpm was subsequently maintained until the end of the culture period. The supernatant was collected for exosome isolation and purification, and the cells were digested with trypsin to determine the cell number and cell viability and for other bioactivity assessments.

Mouse fibroblasts derived from the skin of nude mice were harvested and cultured from primary skin cells obtained from 1‐week‐old nude mice. Euthanized mice were immersed for 5 min in 75% alcohol. The epidermis was carefully removed utilizing surgical scissors and then immersed in phosphate‐buffered saline (PBS) for cleaning. The epidermis was subsequently cut into small squares measuring 1.0 × 1.0 mm, and the dermis was carefully placed in a 6.0 cm^2^ cell culture dish. On the first day, 2 mL of complete culture medium (DMEM (Suzhou Youyi Biotech, China)) supplemented with 50% fetal bovine serum (Suzhou Ecosai Biotech, China) and 1.0% penicillin‒streptomycin (Beyotime, China)) was added to the cell culture dishes, after which the cells were cultured at 37°C in a 5% CO_2_ incubator. On the second day, 6 mL of complete medium (DMEM supplemented with 10% fetal bovine serum and 1.0% penicillin‒streptomycin) was added. When the cells reached 50% confluence, the fibroblasts (Fbs) began to crawl out of the dish and were subsequently digested with 0.125% trypsin (Gibco, USA) to obtain P0 cells, which were used in the subsequent experiments.

Human skin fibroblasts were obtained from the Cell Bank of the Chinese Academy of Sciences (Shanghai, China).

### 2.2 Administration of 3H‐EXO or miRNA on Skin Wound Healing Mouse

White BALB/C‐nu mice (males, 6–8 weeks of age) were purchased from Shanghai Surgeo Technology Company (China). All surgical procedures performed on the animals were approved by the Ethical Review Committee of Shanghai Ruitimos Biotechnology Co., Ltd. (No. 20241015(12)). The maximum permissible trauma wound size was set at 1000 mm^3^, and the wound size or trauma burden did not exceed this limit during the study. All the mice were housed in a temperature‐controlled room (25°C) with free access to food and water. The mice were maintained under a 12‐h light/dark cycle. White BALB/C‐nu mice were acclimated to the environment in a sterile incubator for one week. The mice were anesthetized using isoflurane, after which a 10 mm wound was created on the skin. Skin defect punchers were used to remove normal skin tissue within the wound model circle. Silicone gaskets 15 mm in diameter were fixed around the circular skin defects to prevent the mice from scratching and reduce automatic skin contraction. Afterward, subcutaneous injections of exosomes (100 µL, 50 µg/mL) were administered with a syringe on Days 0, 3, 5, and 7 after wound creation, and the wounds were photographed to record the healing process at Days 0, 3, 7, 10, and 14 after surgery. The mice were euthanized after wound healing for 7 or 14 days. Samples of the tissues surrounding the wounds were obtained and prepared as paraffin sections, which were used for haematoxylin and eosin (H&E) staining, Masson's trichome staining, and immunofluorescence staining of COL‐I/COL‐III and *α*‐smooth muscle actin (*α*‐SMA). The wound healing diameter was measured after H&E staining and calculated using K‐Viewer software (Konfoong Tech, China). Moreover, the wound ratio (%) was calculated according to the following equation:

(1) $$woundratio=A_{C}/A_{O}\;\times \;100\%$$

where A_O_ represents the original area of the wound, and A_C_ represents the current area of the wound.

In addition, 10 nM agomir‐185‐5p, antagomir‐185‐5p, 3H‐EXO, and 3H‐EXO in combination with the antagomir‐185‐5p were administered by subcutaneous injection around the wound margin. Treatments were given on Days 0, 2, 4, and 6, and wound healing was monitored over 14 days. Moreover, the wound tissues were harvested for histological assessment (H&E and Masson staining), quantitative immunofluorescence evaluation, and RT‒qPCR analysis on Day 14.

### 2.3 Isolation and Characterization of Exosomes

Following hUC‐MSC culture, exosomes were isolated from the culture supernatant via centrifugation at 300 × *g* for 10 min, 2000 × *g* for 30 min, and 120,000 × *g* for 2 h. The particle number of the exosomes was determined using a NanoSight Pro (Malvern Panalytical, UK). hUC‐MSC exosome production was assessed as the number of exosome particles produced by a single type of hUC‐MSC. The morphology of the exosomes was observed using transmission electron microscopy (TEM; JEOL, Japan), and surface marker proteins (such as CD63, CD9, ALIX, and TSG101) were identified through western blot (Abcam, UK) and nanoflow cytometry (VDO Biotech, China) analyses. To distinguish the purified exosomes from other extracellular vesicles (EVs), RT‒qPCR and western blot were conducted to determine the gene expression and protein expression, respectively, of CHMP6, a key protein involved in exosome biogenesis.

### 2.4 Flow Cytometry Analysis

On the basis of five negative biomarkers (CD19, CD11b, CD34, CD45, and HLA‐DR) and three positive biomarkers (CD73, CD90, and CD105), the hUC‐MSCs cultured under different conditions were characterized using flow cytometry (CytoFLEX, Beckman, USA). The density of cells in each sample was not less than 10 × 10^6^ cells/mL, and the assay duration was shortened to 2 h to minimize error related to cell viability.

### 2.5 Stem Cell Differentiation Assay

hUC‐MSCs were induced to differentiate into osteogenic, lipogenic, and cartilaginous cells over a period of 2–3 weeks, following the procedures specified by the reagent kits (Absin, China). The cultured cells were identified using staining methods, including alizarin red staining (Beyotime, China) for osteoblasts, oil red O staining (Biosharp, China) for lipoblasts, and alcian blue staining (Merck, Germany) for chondrocytes. The stained samples were observed and recorded under a light microscope (MSX2; Mshot, China).

### 2.6 Cellular Senescence Assay

Lysosomal senescence is an important indicator of cellular senescence. The cells were cultured at a density of 8 – 10 × 10^4^ cells/mL in six‐well plates until they reached 80% confluence. The cells were subsequently washed twice with PBS and stained for 30 min at room temperature in accordance with the instructions of the *β*‐galactosidase kit (Beyotime Biotech, China). The stained samples were observed and recorded under a light microscope (MSX2, Mshot, China).

### 2.7 1 3,5‐Ethynyl‐2′‐deoxyuridine (EdU) Assay for Fibroblast Proliferation

The optimal concentration of exosomes was determined with EdU assays. In brief, fibroblasts (Fbs) were treated with 1, 5, 10, 20, or 50 µg/mL exosomes after 24 h of culture, and EdU reagent (Beyotime Biotech, China) was added after 48 h of culture. 2 h later, the fluorescence intensity of the fibroblasts was observed with a confocal laser scanning microscope (FV4000, OLYMPUS, Japan).

### 2.8 Immunofluorescence Analysis of F‐actin and MitoTracker Red Staining in Fibroblasts

The functional activity of fibroblasts (Fbs) treated with exosomes, including their ability to respond to F‐actin and MitoTracker red staining intensity, was determined. Briefly, after 24 h of Fbs culture, exosomes were added for coculture. After 72 h of coculture, the cells were washed twice with phosphate‐buffered saline (PBS). For F‐actin analysis, Fbs were stained using CoraLite Plus 488 from Proteintech (China) and observed under a confocal laser scanning microscope (FV4000; Olympus, Japan). For MitoTracker red analysis, the Fbs were stained using MitoTracker Dyes for mitochondria labeling, M22426 (Thermo Fisher, US), and observed under a confocal laser scanning microscope (FV4000; Olympus, Japan).

### 2.9 ATP Content Assay of Fibroblasts

ATP content was measured using an ATP Assay Kit (Beyotime, China). The culture medium was removed from the 6‐well plate, 200 µL of cell lysis buffer was added, and the mixture was gently pipetted up and down for 5 s. Next, the lysis buffer was transferred to an EP tube and centrifuged at 12,000 g at 4°C for 5 min. The supernatant was retained, and the working solution was prepared according to the instructions of the kit. Next, 100 µL of working solution was added to the 96‐well plate and incubated at room temperature for 5 min. A 20 µL sample was subsequently added, and the fluorescence intensity was immediately detected (fluorescence spectrophotometer F98, China). Steps were performed on ice when necessary. Additionally, the expression of ATP synthesis–related genes, including PCG‐1*α*, TFAM, NRF1, SOD2, and ATP5a, was quantified in Fbs using RT‒qPCR, as described in detail in the subsequent methods section. To further clarify the relationship between ATP alterations and collagen production, collagen‐related gene expression was examined in Fbs cultured under serum‐free low‐glucose conditions (DMEM, 1 g/L). After an initial 24‐h culture period, the cells were transferred to serum‐free medium for an additional 12 h. Subsequently, 3H‐EXO and normal medium were added to the exosome group and the ATP rescue group, respectively, for 12 h of culture. The cultured cells were digested with trypsin, after which RT‒qPCR detection of COL‐I and COL‐III was performed. In parallel, serum‐free medium supplemented with 3H‐EXO was used.

### 2.10 Transcriptomic of Fibroblasts

After 24 h of coculture with exosomes, the Fbs were collected for transcriptomic analysis. RNA transcriptome sequencing and analysis were conducted by OE Biotech (Shanghai, China). Data processing was performed on the OE Biotech cloud platform (https://cloud.oebiotech.cn/#/bio/tools?cat_id = &tag_id = &search =). The raw transcriptome sequencing data are available in the Sequence Read Archive database (https://submit.ncbi.nlm.nih.gov/subs/bioproject/SUB15633713/overview) with the accession number PRJNA1327593.

### 2.11 RT‒qPCR Analysis

Briefly, an RNA/DNA Isolation Kit (Beyotime, China) was used to isolate RNA from Fbs, ABScript III RT Master Mix for qPCR with gDNA Remover (ABclonal, China) was used to reverse transcribe Fbs RNA into cDNA, and qPCR master mix (Vazyme, China) was used to quantify gene levels. The RT‒qPCR primer sequences are shown in Table S1. After skin fibroblasts were cultured for 24 h, 50 nM miR‐185‐5p mimic, antagomir‐185‐5p, 3H‐EXO, or 3H‐EXO combined with the antagomir‐185‐5p was added to the cells, followed by an additional 48 h of incubation. A subset of the treated cells was subjected to immunofluorescence staining to evaluate the expression of COL‐I, COL‐III, and *α*‐SMA. The remaining cells were collected for RT‒qPCR analysis of the expression of RhoA, YAP, TGF‐*β*1, and SMAD7. An acute wound model in mice was established following a previously described protocol. The miRNAs were extracted from the ultracentrifugation‐purified exosome fractions using a commercial exosome miRNA extraction kit (Absin, China). Specific primers for miR‐185‐5p, miR‐423‐5p, miR‐320a‐5p, and let‐7b‐5p were prepared and diluted to a final concentration of 10 nM with RNase‐free water. qPCR amplification was performed using miRNA Unimodal SYBR qPCR Master Mix (Vazyme, China) according to the manufacturer's instructions. miRNA samples derived from exosomes obtained under different culture conditions were analyzed sequentially to quantify their expression levels.

### 2.12 Western Blot Analysis

To prepare samples for WB, prechilled lysates were added to Fbs, followed by intermittent sonication for 1.0 min and centrifugation in a cryogenic centrifuge at 4°C for 30 min at 500 × *g* to obtain the proteins in the supernatant. Moreover, the protein concentration was determined using a BCA protein concentration assay kit (Beyotime, China). Afterward, a Yeasen Fast Gel Kit (China) was used to prepare 10% gels, and the denatured proteins were loaded with 5 × loading buffer (Beyotime, China) and separated for 2 h at 80 V. Protein transfer was performed by attaching the protein gel to a polyvinylidene fluoride (PVDF) membrane for 1.0 h at 110 V, after which the PVDF membrane was immersed in skim milk for 2 h at room temperature, followed by incubation with the primary antibody for 12 h at 4°C. Finally, the membrane was incubated with secondary antibody for 2 h at room temperature, after which the protein was visualized using enhanced chemiluminescence (ECL; Yeasen Biotech, China). All protein antibody information is shown in Table S2.

### 2.13 Function of the RhoA/YAP Pathway in Fibroblasts

To determine the function of the RhoA/YAP pathway in fibroblasts, a RhoA inhibitor, a YAP inhibitor, a YAP activator (PY‐60, MCE, China), and exosomes were added to fibroblasts. After 24 h of coculture, the cells were collected for WB and RT‒qPCR analysis of key factors such as COL‐I, COL‐III, YAP, and RhoA.

### 2.14 Luciferase Reporter Assay for the Target Genes miR‐185‐5p and RhoA

The binding sites between miR‐185‐5p and RhoA were predicted using the database miRDB. Subsequently, miR‐185‐5p mimic and inhibitors were synthesized. The wild‐type (RhoA‐NC) and mutant (RhoA‐MU) 3′‐UTR sequences of RhoA were cloned and inserted into firefly luciferase reporter plasmids, after which the RhoA‐NC, RhoA‐MU, miR‐185‐5p mimic, and negative control (miR‐NC) plasmids were transfected into HEK‐293 cells using Lipofectamine 3000 (Thermo Fisher Scientific, USA) for 48 h. Afterward, the cells were collected and lysed with lysis buffer. Finally, luciferase activity was measured using a Dual‐Luciferase Reporter Assay Kit (Novezyme, China).

### 2.15 Statistical Analysis

Statistical analysis was performed using ImageJ software (version 13.0.6) and GraphPad Prism software (version 10.2.3). Significant differences between groups were analyzed using *t*‐tests. Two‐way analysis of variance (ANOVA) was used to assess the statistical significance of the mean values of more than two groups. All the experimental results were acquired from at least three biological replicates. The data are presented as the mean ± SD; *p < 0.05, **p < 0.01, ***p < 0.001, ****p < 0.0001. A *p* value less than 0.05 was considered to indicate statistical significance.

## 3 Results

### 3.1 High Performance of hUC‐MSCs Grown in a 3D Suspension Culture

2D cultures of hUC‐MSCs were generated in different O_2_ atmospheres (21%, 7%, 5%, 3%, and 1%). The cell number, glucose concentration, and lactate concentration of each culture are shown in Figure S1. The results revealed that there was no significant difference between the 21% and 7% O_2_ groups. However, at an oxygen content of 5%, a decrease in cell number was observed, followed by no cell growth at oxygen concentrations of 3% and 1%. These findings suggest that 5% oxygen should be considered a critical threshold for the growth of hUC‐MSCs. Under these conditions, cells can sense changes in the microenvironment and respond accordingly without completely halting growth. Consequently, 5% oxygen was used for subsequent experiments involving hypoxic culture.

**FIGURE 2:**
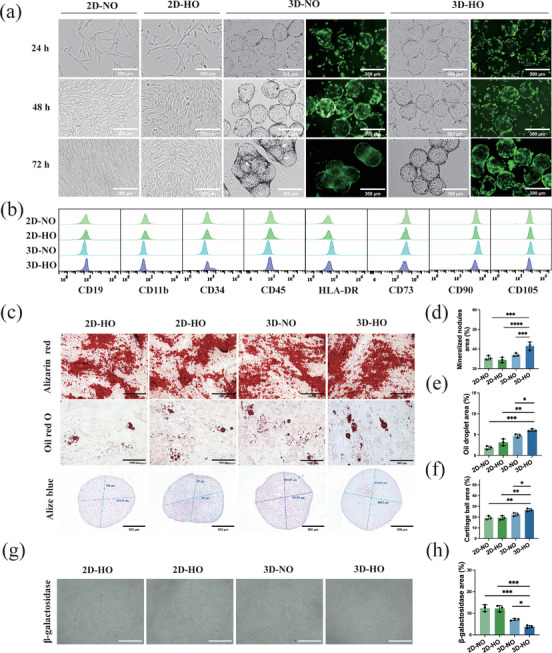
Effects of different culture conditions on the growth characteristics and stemness of stem cells (a) Morphological structures of stem cells at different culture times and different conditions (grey: bright field; green: fluorescence). (b) Flow cytometry analysis of UC‐MSC characteristics. (c) Microscopy images of UC‐MSCs after osteogenic, lipogenic, and chondrogenic differentiation. (d–f) Statistical analysis of the osteogenic (scale bar = 1000 µm), lipogenic (scale bar = 1000 µm), and chondrogenic (scale bar = 300 µm) differentiation of stem cells. (g) Senescence (*β*‐galactosidase) of UC‐MSCs (scale bar = 1000 µm). (h) Statistical analysis of *β*‐galactosidase expression. The data are presented as the mean ± SD; *p < 0.05, **p < 0.01, ***p < 0.001, ****p < 0.0001.

The characteristics of the hUC‐MSCs grown in flasks for 2D culture and 3D suspension culture are illustrated in Figure 2a and Figure S2. All the cells exhibited favorable growth at various stages with high viability. The stemness of the hUC‐MSCs was subsequently assessed by evaluating the expression of specific surface marker proteins (Figure 2b), with more than 96% of the cell population expressing the three positive markers CD105, CD90, and CD73. Moreover, less than 2.0% of the cell population expressed five negative marker proteins, namely, CD19, CD11b, CD45, CD34, and HLA‐DR, thus fulfilling the criteria for stem cells. Furthermore, the multidirectional differentiation potential of hUC‐MSCs into osteogenic, lipogenic, and chondrogenic lineages (Figure 2c) was noted for cells grown in 3D hypoxia culture (3D‐HO), which also produced more mineralized nodules (Figure 2d), larger and more abundant lipid droplets (Figure 2e), and larger cartilage spheres (Figure 2f) than those grown in 2D normal oxygen (2D‐NO) culture did. The results of the CCK8 assay revealed no significant difference in the cell survival rate between the 5% oxygen concentration and normoxic culture (Figure S3). Additionally, the senescence of hUC‐MSCs cultured under conditions was analyzed by measuring the expression of the marker *β*‐galactosidase (Figure 2g,h), and the results indicated that 3D culture significantly reduced *β*‐galactosidase production and inhibited cell ageing. Therefore, 3D suspension culture and hypoxic stimulation promoted an improvement in hUC‐MSC stemness and the maintenance of cell viability.

### 3.2 Characterization of Exosomes Derived From hUC‐MSCs Grown in 3D Hypoxic Suspension Culture

**FIGURE 3:**
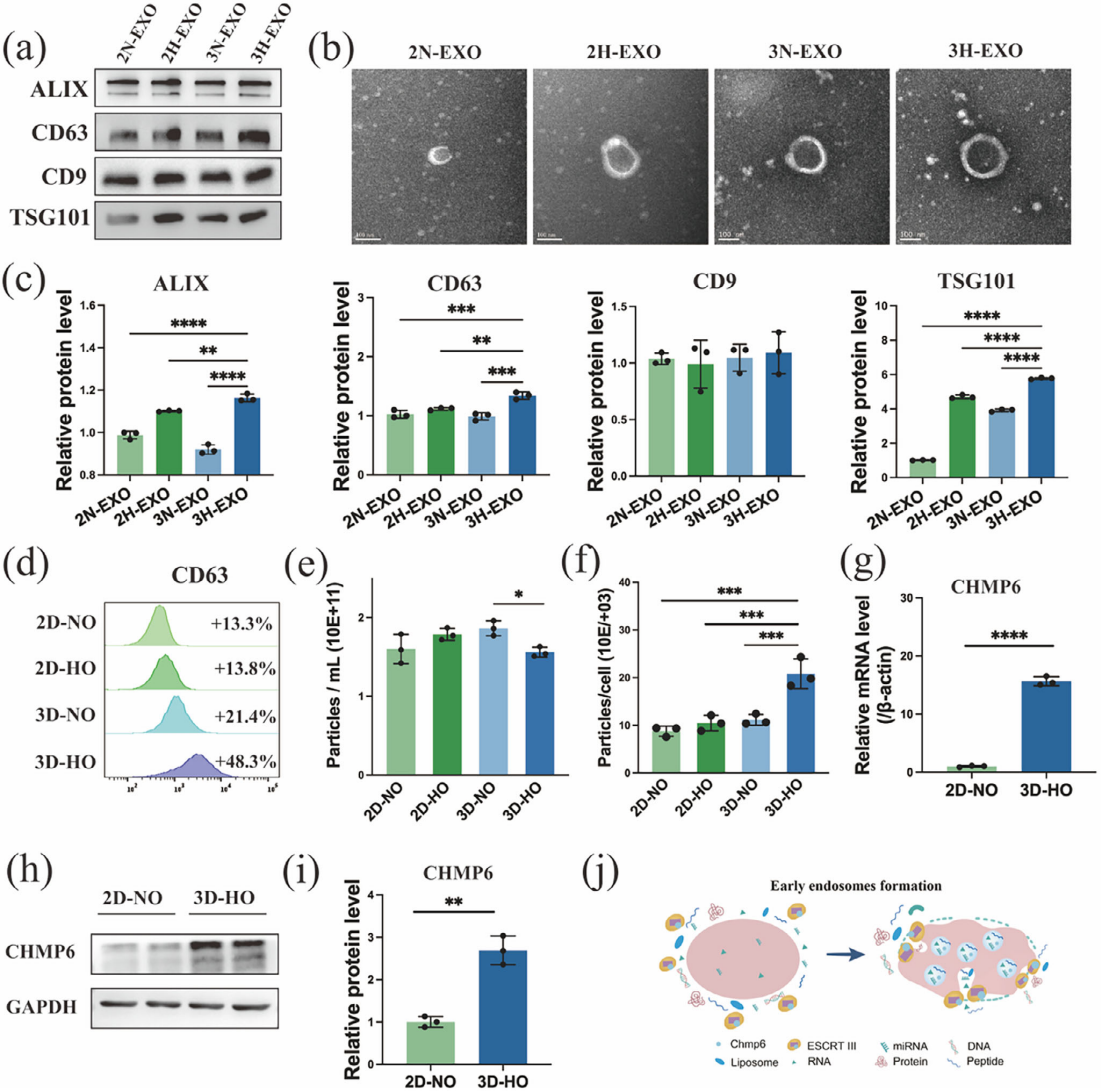
Effects of different culture conditions on stem cell exosome formation. (a) Western blot of exosome marker proteins. (b) Morphological characteristics of the exosomes observed by transmission electron microscopy (TEM; scale bar = 100 µm). (c) Statistical analysis of the Western blot results. (d) Nanoflow cytometry analysis of exosomes (CD63). (e) Exosome particles produced under different culture conditions were analyzed by nanoparticle tracking analysis (NTA). (f) The average number of exosomes produced per cell under different culture conditions. (g) Levels of genes encoding CHMP6 (an exosome marker protein) determined by RT‒qPCR. (h) Levels of CHMP6 (an exosome marker protein) determined by Western blot. (i) Statistical analysis of CHMP6 expression by Western blot. (j) Schematic diagram of exosomes formed by the ESCRT pathway. The data are presented as the mean ± SD; *p < 0.05, **p < 0.01, ***p < 0.001, ****p < 0.0001.

The exosomes secreted from hUC‐MSCs cultivated under different oxygen stimulation conditions were purified and characterized, as shown in Figure 3. WB (Figure 3a,c) revealed significantly increased levels of the specific positive biomarkers CD9, CD63, ALIX, and TSG101 in 3H‐EXOs compared with those in exosomes from the other groups. The 3H‐EXOs exhibited a more distinct and streamlined teacup‐like structure according to transmission electron microscopy (TEM) (Figure 3b). Furthermore, many extracellular vesicle particles were identified through nanoparticle tracking analysis (NTA) (Figure 3e; Figure S4). Moreover, the average number of exosome particles per cell (particles/cell) was used to evaluate exosome production by hUC‐MSCs. The results demonstrated that exosome production in 3D‐HO culture was 2.43 times, 2.01 times and 1.88 times greater than that in 2D‐NO, 2D‐HO, and 3D‐NO culture, respectively (Figure 3f). Additionally, nanoflow cytometry analysis of exosomes expressing CD63 (Figure 3d) revealed that the expression level of CD63 was the highest in the 3D‐HO group, indicating that more exosomes were present in the same number of particles. Furthermore, RT‒qPCR (Figure 3g) and WB (Figure 3h,i) analyses of the expression of CHMP6, a key protein involved in exosome biogenesis (Figure 3j), were performed to confirm the increased abundance of exosome synthesis proteins in the 3D‐HO group at the gene and protein levels. Overall, these findings support that the hUC‐MSCs in the 3D‐HO group have the potential to generate more EVs, with a significant increase in the number and proportion of exosomes, not just vesicles.

### 3.3 Exosomes Derived From hUC‐MSCs in 3D‐HO Effectively Facilitate Scarless Wound Healing in Mice

**FIGURE 4:**
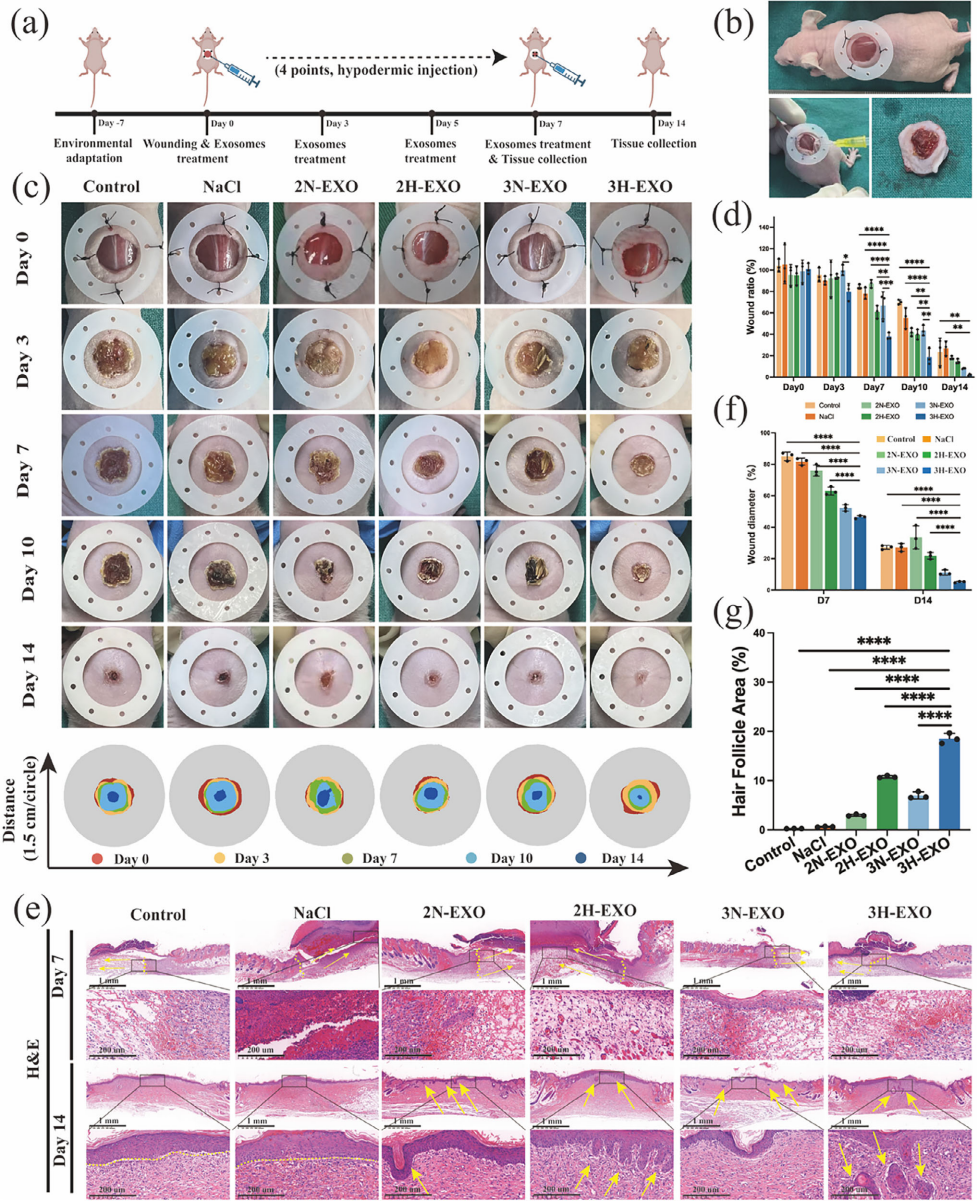
3H‐EXOs effectively promote rapid scarless wound healing in mice. (a) Schematic diagram of the mouse wound model and exosome treatment. (b) Image of the mouse wound treatment process. (c) Photographic images of wound recovery in mice (*n* = 3). (d) Statistical analysis of the wound healing area during recovery in the mice (*n* = 3). (e) H&E staining images of mice wound tissue (*n* = 3; black square: junction between the wound and normal skin; yellow area: wound tissue; scale bar = 1 mm, 200 µm). (f) The wound diameter of the mice was calculated based on the H&E staining images. (g) Wound area with hair follicle growth after 14 days (*n* = 3). The data are presented as the mean ± SD; *p < 0.05, **p < 0.01, ***p < 0.001, ****p < 0.0001. Control: no treatment; NaCl: NaCl treatment; 2N‐EXO: exosomes from 2D culture under normal oxygen conditions; 2H‐EXO: exosomes from 2D culture under hypoxic conditions; 3N‐EXO: exosomes from 3D culture under normal oxygen conditions; 3H‐EXO: exosomes from 3D culture under hypoxic conditions.

Mice with acute wounds were treated with exosome injections following the method outlined in Figure 4a,b, and images of wound healing at various time points are presented in Figure 4c. The morphology of the wounds in all the groups was clear on Days 0, 3, 7, and 10. However, compared with those in the control group, the wounds in the 3H‐EXO group exhibited the earliest signs of scarless healing on Day 14 (Figure 4d–f, *p* < 0.0001). In contrast, the other groups continued to exhibit prominent scab layers on Day 14. Haematoxylin and Eosin (H&E) staining (Figure 4e) revealed that a dense extracellular matrix (ECM) formed between the wound margins and normal skin in the 3H‐EXO group first, which was due to the remodeling and cross‐linking of different types of collagens (COL‐I and COL‐III). Furthermore, compared with the control group, the hypoxic exosome group was more capable of generating new hair follicle structures (Figure 4g).

**FIGURE 5:**
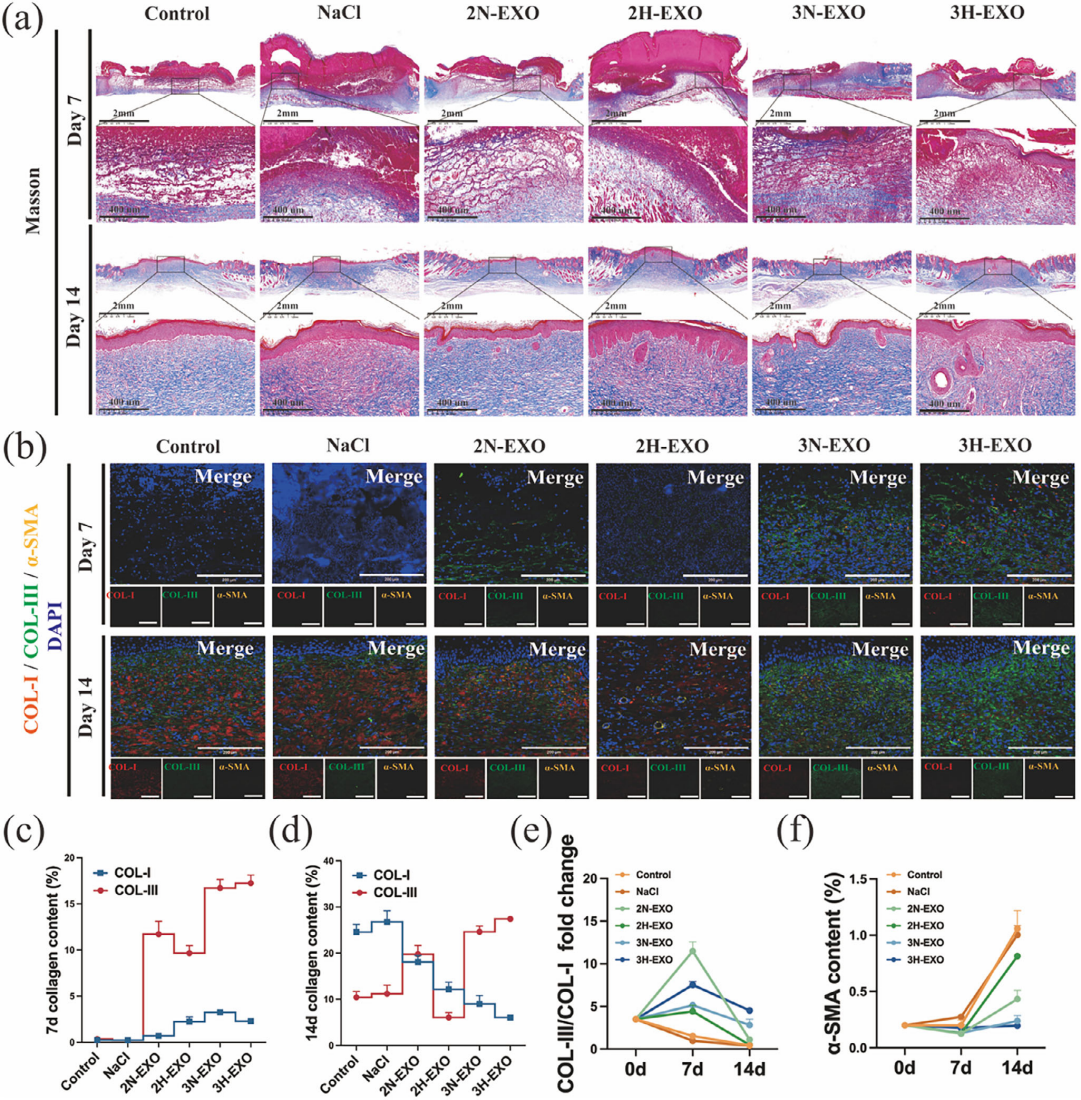
Effects of exosomes on total collagen formation (Masson staining) and the formation of different collagen types (COL‐I/COL‐III) during mouse wound healing. (a) Masson staining image of the mice wound tissue (*n* = 3; scale bar = 2 mm; 400 µm). (b) Immunofluorescence staining of COL‐I, COL‐III and *α*‐SMA in mice wound tissue (*n* = 3; scale bar = 200 µm). (c–f) Statistical analysis of the fold changes in COL‐I and COL‐III expression and the COL‐III/COL‐I ratio and *α*‐SMA immunofluorescence staining. Control: no treatment; NaCl: NaCl treatment; 2N‐EXO: exosomes from 2D culture under normal oxygen conditions; 2H‐EXO: exosomes from 2D culture under hypoxic conditions; 3N‐EXO: exosomes from 3D culture under normal oxygen conditions; 3H‐EXO: exosomes from 3D culture under hypoxic conditions. COL‐I: collagen Type I; COL‐III: collagen Type III.

These results indicate that the wounds of mice in the 3H‐EXO group exhibit good wound healing ability in terms of both ECM remodeling and hair follicle regeneration. The results of Masson staining (Figure 5a) also demonstrated that 3H‐EXOs are capable of increasing collagen levels on both Day 7 and Day 14 (Figure S5). However, Masson staining reflects the total amount of collagen, including both Type I and Type III collagens. It is necessary to determine the types of collagen that play positive roles in 3H‐EXO‐mediated wound healing. As shown in Figure 5b, the COL‐I and COL‐III levels tended to change during the wound healing process. A comparison of the changes in wound healing on Day 7 revealed that compared with COL‐I, COL‐III exhibited a more rapid growth pattern (Figure 5c). The COL‐III content in the 3D culture group was significantly greater than that in the 2D culture and control groups on both Day 7 and Day 14. However, a significant decrease in the COL‐I level was observed on Day 14 compared with that in the control group (Figure 5d). Additionally, a statistical analysis of the COL‐III/COL‐I ratio (Figure 5e) in the 3H‐EXO group revealed a ratio of 4:1 on Day 14. Although the collagen ratio of the exosome groups tended to initially increase but then decrease, only the 3D culture group consistently maintained a higher COL‐III content than COL‐I content during the latter stages of wound healing. *α*‐Smooth muscle actin (*α*‐SMA) serves as a principal marker of myofibroblasts (myoFbs) [35], the predominant cells responsible for the secretion of COL‐I during the process of wound healing. In the tissues of the mice on Day 7, there was no significant difference in *α*‐SMA levels between the groups (Figure 5f). However, *α*‐SMA expression was significantly elevated in both the control and 2D culture groups on Day 14. These findings suggest that 3H‐EXOs can decrease the overexpression and formation of COL‐I by inhibiting the activation of myofibroblasts. These results demonstrate that the 3H‐EXOs of hUC‐MSCs promote scarless wound healing in mice by precisely regulating the COL‐I to COL‐III ratio during regeneration.

### 3.4 Effect of 3H‐EXOs on the Physiological Activity of Mouse Fibroblasts

**FIGURE 6:**
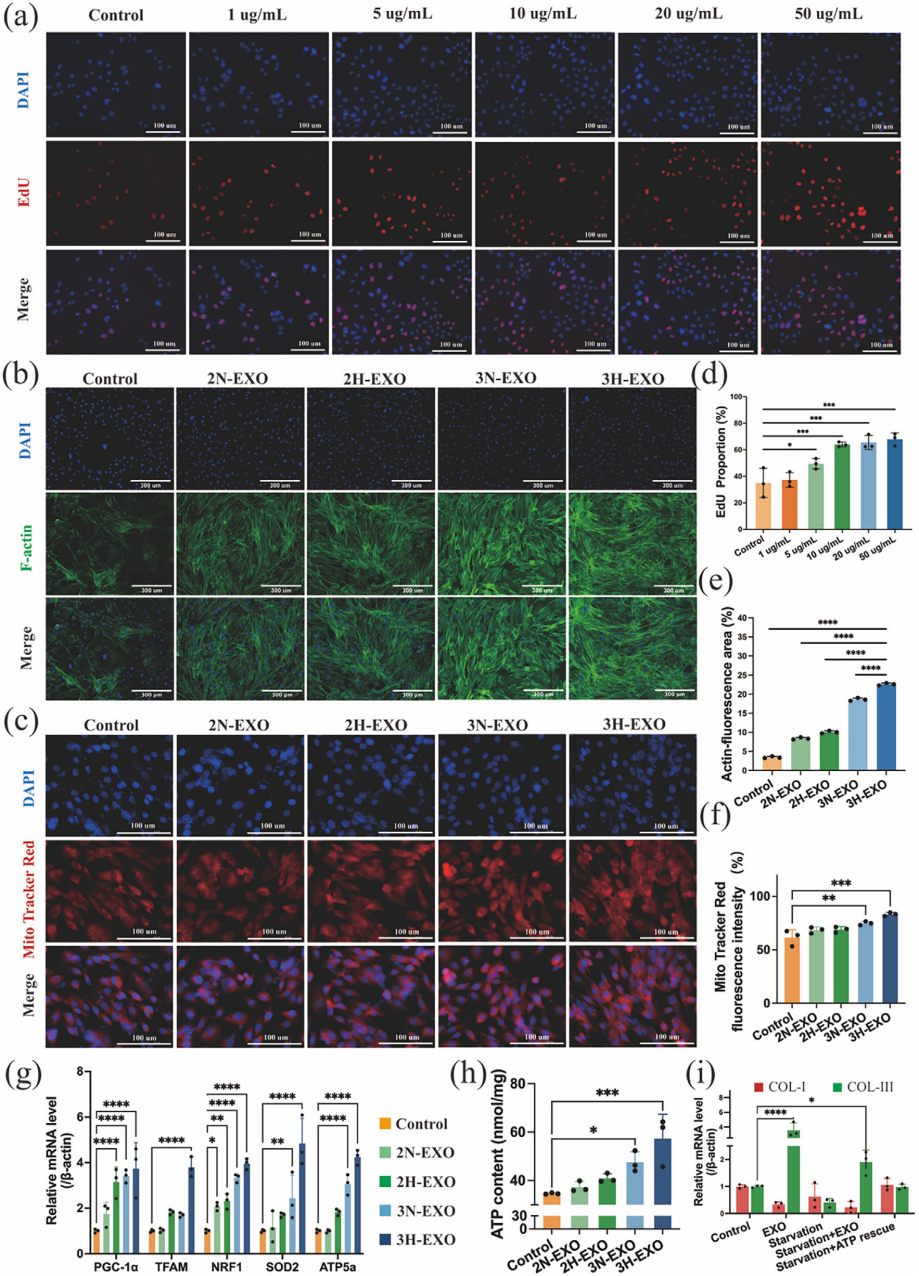
Effects of exosomes on the proliferation and function of mouse fibroblasts. (a) Effects of treatment with different concentrations of 3H‐EXOs on the proliferation of fibroblasts at 2 h (*n* = 3; scale bar = 100 µm). (b) Effect of exosomes on F‐actin formation in fibroblasts (*n* = 3; scale bar = 300 µm). (c) Effect of exosomes on MitoTracker Red staining of fibroblasts (*n* = 3; scale bar = 100 µm). (d) Statistical analysis of the proliferation rate at 2 h determined by an EdU assay. (e) Statistical analysis of F‐actin fluorescence intensity. (f) Statistical analysis of MitoTracker Red fluorescence intensity. (g) mRNA levels of genes related to ATP synthesis. (h) Changes in ATP content after fibroblasts were treated with different types of exosomes. (i) The influence of low ATP interference on collagen synthesis. The data are presented as the mean ± SD; *p < 0.05, **p < 0.01, ***p < 0.001, ****p < 0.0001. Control: no treatment; NaCl: NaCl treatment; 2N‐EXO: exosomes from 2D culture under normal oxygen conditions; 2H‐EXO: exosomes from 2D culture under hypoxic conditions; 3N‐EXO: exosomes from 3D culture under normal oxygen conditions; 3H‐EXO: exosomes from 3D culture under hypoxic conditions.

Endocytosis of PKH26‐labeled 3H‐EXOs (Figure S6a) and the effects of 2D migration on fibroblasts (Fbs) are shown in Figure S6b. The results demonstrated that the exosomes could be endocytosed within 30 min after administration and exhibited significant cell planar migration after 24 h. Moreover, on the basis of the proportion of EdU‐positive cells (Figure 6a), the administration of 50 µg/mL exosomes had the most significant effect on proliferation after 2 h of treatment, with a 1.94‐fold increase compared with that in the control group (Figure 6d). Furthermore, F‐actin is another core indicator involved in cell migration, division and proliferation. It is a long‐chain microfilament formed by the aggregation of G‐actin and serves as the core structure of the cytoskeleton to maintain cell morphology. F‐actin fluorescence staining of fibroblasts treated with different types of exosomes is shown in Figure 6b. The results shown in Figure 6e indicate that 3H‐EXO treatment promotes an increase in F‐actin, suggesting that the cells are in an active growth or movement state. This phenomenon was indicated by the fluorescence intensity of G‐actin, which decreased relative to that in the other groups following 3H‐EXO treatment (Figure S7a,b) and maintained a dynamic balance with that of F‐actin in the ECM. To further assess whether these changes are associated with alterations in mitochondrial function, the mitochondrial membrane potential was determined using MitoTracker, and the content of mitochondrial‐related genes and the total ATP content of the cells were determined. Figure 6c–f suggest that 3D culture of exosomes increased the membrane potential, with a more significant effect in the hypoxia group. This finding is consistent with the ATP content results (Figure 6h). Moreover, the expression of the genes PGC‐1*α*, TFAM, NRF1 and ATP5A, which are related to mitochondrial synthesis and function in cells (Figure 6g), significantly improved after 3H‐EXO treatment, and the activity of the antioxidant enzyme SOD2 particularly improved. Moreover, a reduction in the ROS content (Figure S7c,d) indicates that mitochondria may produce more ATP. Since ATP serves as a crucial energy source for collagen synthesis and fibroblast activation, its effect on collagen expression was determined through low‐ATP intervention (Figure S7e). Figure 6i shows the overall inhibitory effect of ATP restriction on collagen after starvation stimulation. With the addition of EXO and the recovery of ATP, only EXO has the same regulatory effect in both energy states. On the basis of the above results, 3H‐EXOs were shown to increase cellular homeostasis upon internalization, including increased ATP production, increased proliferative capacity, and enhanced cytoskeletal remodeling. However, these global improvements in cellular vitality alone are insufficient to account for the observed shift in collagen subtype proportions. Instead, the directional change in collagen composition is likely driven by specific signaling molecules—particularly miRNAs—packaged within the exosomes.

### 3.5 Mechanism Through Which 3H‐EXOs Regulate Collagen III/I Secretion via the RhoA/YAP Pathway

**FIGURE 7:**
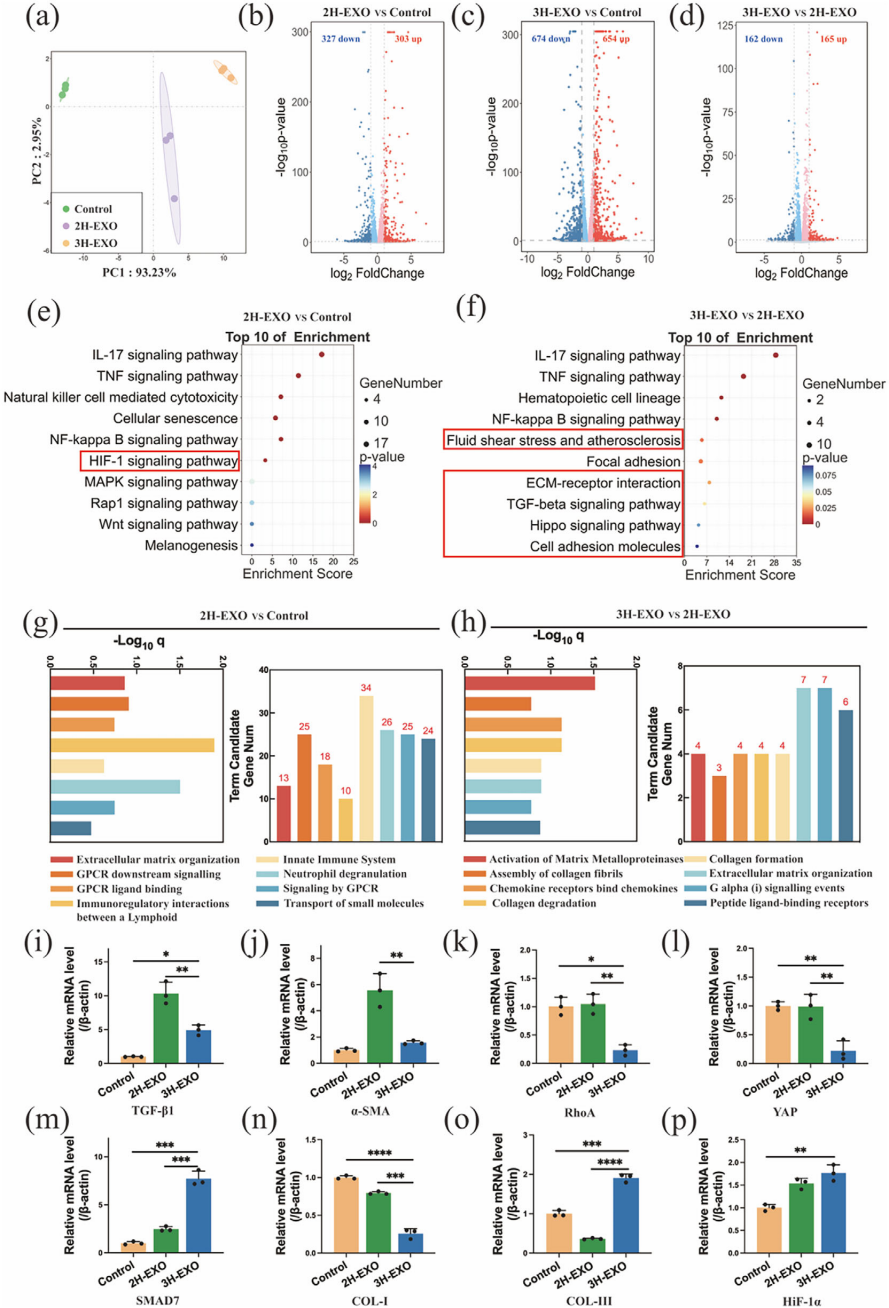
RNA sequencing analysis of fibroblasts treated with exosomes. (a) Principal component analysis (PCA) diagram showing the variance and standard deviation of the control, 2H‐EXO, and 3H‐EXO datasets (*n* ≥ 3). Volcano plots displaying differential gene expression profiles for the (b) 2H‐EXO group versus the control group, (c) 3H‐EXO group versus the control group, and (d) 3H‐EXO group versus the 2H‐EXO group. Red (FC > 1.5) and blue (FC < 1.5) data points represent genes with the indicated fold change in expression and a false discovery rate (FDR) < 0.05. Functional overrepresentation analysis using differentially expressed genes for the 2H‐EXO versus control and 3H‐EXO versus 2H‐EXO comparison groups with the (e, f) KEGG and (g, h) Reactome databases. Statistical analysis of (i) TGF‐*β*1, (j) *α*‐SMA, (k) RhoA, (l) YAP, (m) SMAD7, (n) COL‐I, (o) COL‐III, and (p) HiF‐1*α* gene expression determined via RT‒qPCR. The data are presented as the mean ± SD; *p < 0.05, **p < 0.01, ***p < 0.001, ****p < 0.0001.

In vivo and in vitro experiments demonstrated that 3H‐EXO preferentially promoted collagen synthesis in fibroblasts (Fbs) (Figures 5 and 6). To explore the mechanism through which exosomes regulate collagen formation in Fbs, Fbs treated with exosomes were collected for RNA sequencing, WB, and RT‒qPCR analysis (Figure 7). RNA sequencing of the control, 2H‐EXO, and 3H‐EXO groups was performed. Briefly, principal component analysis (PCA) of the results from the three groups (Figure 7a) revealed high consistency and repeatability. Compared with the other comparison groups, the 3H‐EXO/Control comparison group presented a greater number of differentially expressed genes (Figure 7b–d) with different functions. Compared with the 2H‐EXO/Control comparison group (Figure 7e), the 3H‐EXO/2H‐EXO group (Figure 7f) demonstrated increased enrichment of differentially expressed genes associated with extracellular matrix synthesis, deposition, binding to cell surface receptors, fluid shear stress‐related pathways, and fibrotic disease‐related pathways. In addition, the Reactome database emphasizes the interactions among biomolecules within the pathway and KEGG pathways. The genes that were differentially expressed between the 2H‐EXO and control groups (Figure 7g) and between the 3H‐EXO and 2H‐EXO groups (Figure 7h) were related to the regulation of matrix metalloproteinases (collagen‐degrading enzymes), as well as the synthesis, assembly, and degradation of collagen fibers. On the basis of the results of the KEGG and Reactome analyses, the TGF‐*β* signaling pathway and the Hippo signaling pathway were regarded as the key signaling pathways directly associated with collagen synthesis in the 3H‐EXO group.

**FIGURE 8:**
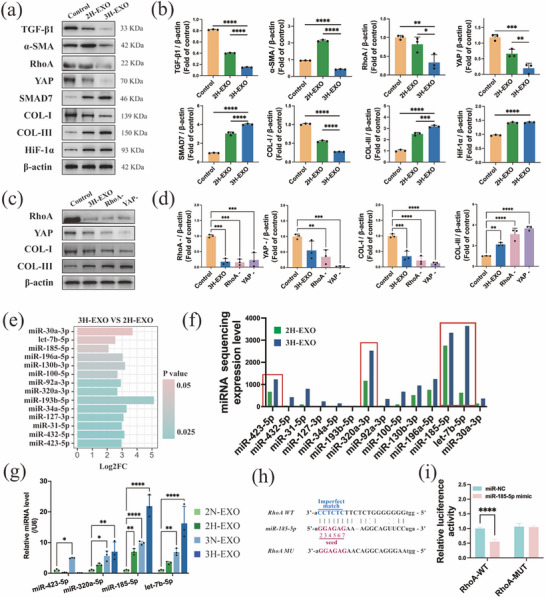
Scarless wound healing in mice mediated by exosomes occurs via the regulation of key factors. Western blots (a) and statistical analysis (b) of TGF‐*β*1, *α*‐SMA, RhoA, YAP, SMAD7, COL‐I, COL‐III, and Hif‐l*α* expression in fibroblasts treated with exosomes. Western blots (c) and statistical analysis (d) of RhoA, YAP, COL‐I, and COL‐III expression in fibroblasts treated with a RhoA inhibitor or a YAP inhibitor. (e) Statistical analysis of the expression of significant miRNAs in exosomes. (f) miRNA expression levels in the different exosome groups. (g) Relative expression levels of miR‐423‐5p, miR‐320a‐5p, miR‐185‐5p and let‐7b–5p under different culture conditions. (h) Schematic diagram of the potential target binding site between miR‐185‐5p and mouse RhoA. (i) Results of the miR‐185‐5p and RhoA luciferase reporter gene assays. The data are presented as the mean ± SD; *p < 0.05, **p < 0.01, ***p < 0.001, ****p < 0.0001.

To identify how exosomes affect the regulation of both the TGF‐*β* pathway and the Hippo pathway in Fbs, all the key factors involved in these pathways were quantitatively analyzed by RT‒qPCR (Figure 7i–p) and WB (Figure 8a,b). The results demonstrated that the 3H‐EXO group had significantly reduced levels of RhoA, YAP, COL‐I, TGF‐*β*1, and *α*‐SMA but increased levels of SMAD7 and COL‐III. Notably, YAP is a common factor involved in the Hippo and TGF‐*β* signaling pathways and is regulated upstream by RhoA proteins. Furthermore, WB analysis (Figure 8c,d) of Fbs treated with RhoA and YAP inhibitors revealed that compared with those in the 3H‐EXO group, COL‐I levels increased and COL‐III levels decreased. These findings suggest that both RhoA and YAP may be involved in inhibiting COL‐I formation and promoting COL‐III production.

The above results revealed that exosomes precisely regulated the ratio of COL‐III/COL‐I generated by Fbs through the RhoA/YAP pathway. To further explore the key molecules that play a role in exosomes, a total of 14 miRNAs were identified as significantly dysregulated between 3H‐EXOs and 2H‐EXOs (Figure 8e), among which miR‐423‐5p, miR‐320a‐5p, miR‐185‐5p, and let‐7b‐5p were the most abundant in the sequencing dataset (Figure 8f). Subsequent quantification of these candidate miRNAs in exosomes derived from different culture conditions (Figure 8g) revealed that 3D culture markedly enhanced their enrichment, with hypoxic stimulation exerting an additional synergistic effect. Furthermore, in vitro functional evaluations of the four highly expressed miRNAs were conducted in fibroblasts (Figure S8a,b). Compared with miR‐185‐5p, the other three miRNAs did not have obvious collagen regulatory effects. Collectively, these findings indicate that 3D‐HO culture promotes the generation of functionally enriched exosomes and contributes to improved wound healing outcomes.

### 3.6 Mechanism Through Which the miR‐185‐5p‐RhoA/YAP Signaling Axis Promotes Scarless Wound Healing

**FIGURE 9:**
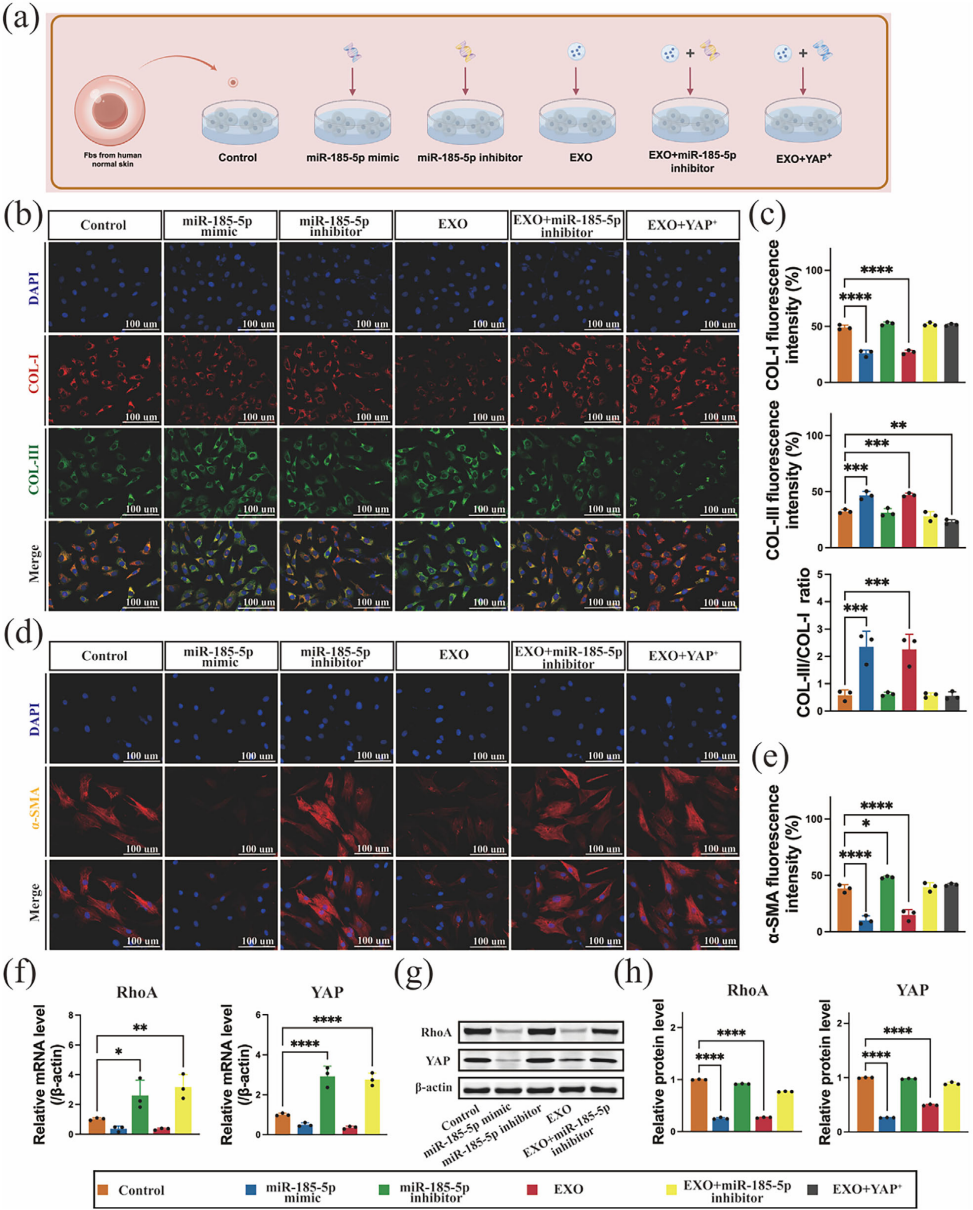
Effect of in vitro inhibition of collagen synthesis by miRNA. (a) Schematic diagram of the role of the miR‐185‐5p‐RhoA/YAP signaling axis in regulating the collagen ratio in fibroblasts. (b) The effect of miR‐185‐5p inhibition on COL‐III/COL‐I in fibroblasts (*n* = 3; scale bar = 100 µm). (c) Statistical analysis of COL‐I and COL‐III fluorescence intensity and the COL‐III/COL‐I ratio. (d) Effect of miR‐185‐5p inhibition on *α*‐SMA expression in fibroblasts (*n* = 3; scale bar = 100 µm). (e) Statistical analysis of *α*‐SMA fluorescence intensity. (f) mRNA levels of RhoA and YAP in human fibroblasts by miR‐185‐5p inhibitor. (g) The protein levels of RhoA and YAP in human fibroblasts by miR‐185‐5p inhibitor. (h) Statistical analysis of RhoA and YAP protein levels. The data are presented as the mean ± SD; *p < 0.05, **p < 0.01, ***p < 0.001, ****p < 0.0001.

The ability of miR‐185‐5p to target the RhoA gene was subsequently verified by a dual luciferase reporter assay (Figure 8h). Compared with the fluorescence intensity in the RhoA‐MUT luciferase group, the fluorescence intensity of luciferase in the RhoA‐WT group decreased because of binding to RhoA (Figure 8i), indicating targeting. Moreover, a miR‐185‐5p mimic and inhibitor were transfected into human skin Fbs, which were then cocultured for 48 h, as shown in Figure 9a. The results of immunofluorescence quantitative analysis (Figure 9b,c) of COL‐I and COL‐III in Fbs revealed that the miR‐185‐5p mimic and EXO groups showed the same trend in regulating COL‐I and COL‐III, whereas the miR‐185‐5p inhibitor group alone showed the opposite trend. This finding indicates that miR‐185‐5p is involved in collagen regulation. Furthermore, the reduction in *α*‐SMA (Figure 9d,e) in the miR‐185‐5p and EXO groups further verified the inhibitory effect of the miR‐185‐5p on COL‐I. Given that miR‐185‐5p targets RhoA, mimic‐ and inhibitor‐based assays support an essential role of RhoA in collagen regulation. Moreover, the results of coculture with a YAP activator corroborated the contribution of YAP signaling to collagen subtype formation. Specifically, YAP activation induced marked increases in COL‐I and *α*‐SMA levels compared with miR‐185‐5p mimic or exosome treatment. Moreover, the corresponding downstream gene levels of RhoA and YAP also changed to varying degrees. In terms of RhoA and YAP mRNA expression levels (Figure 9f), both the EXO and miR‐185‐5p mimic groups tended to decrease, demonstrating the regulatory effect of miR‐185‐5p on RhoA/YAP activity in cells. In addition, miR‐185‐5p inhibits the expression of RhoA/YAP protein levels in human fibroblasts, as shown in Figure 9g,h, showing the same trend as mRNA expression.

**FIGURE 10:**
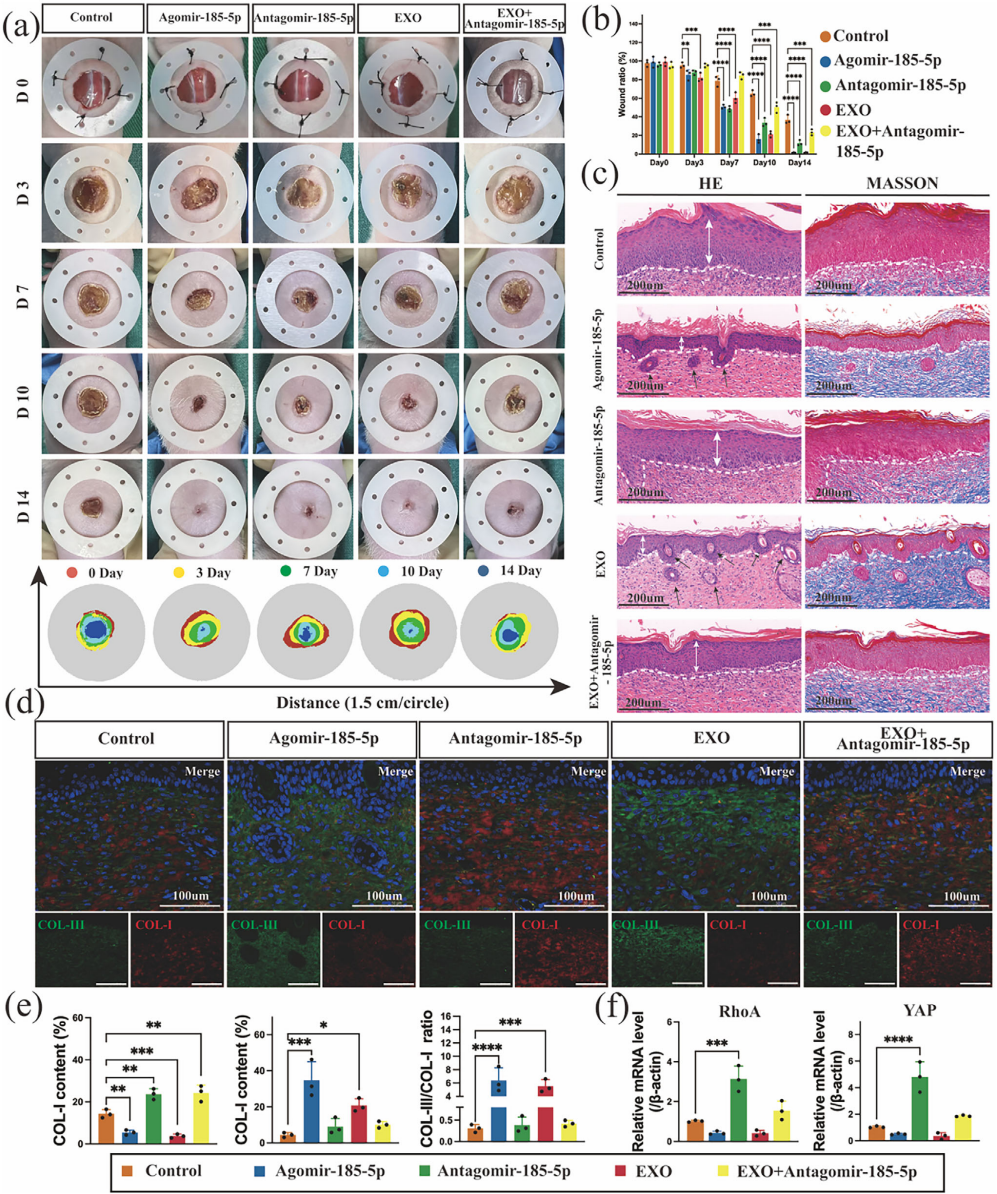
Effect of in vivo inhibition of collagen synthesis by miRNA. (a) Photographic images of wound recovery in mice (*n* = 3). (c) H&E and Masson staining images of mouse wound tissue (*n* = 3; time = 14 days). (d) Immunofluorescence staining of COL‐I and COL‐III in mouse wound tissue (*n* = 3; time = 14 days; scale bar = 100 µm). (b) Statistical analysis of the wound healing ratio during recovery in the mice (*n* = 3). (e) Statistical analysis of COL‐I and COL‐III fluorescence intensity and the COL‐III/COL‐I ratio. (f) Effect of miR‐185‐5p inhibition on the expression of genes related to RhoA/YAP mRNA. The data are presented as the mean ± SD; *p < 0.05, **p < 0.01, ***p < 0.001, ****p < 0.0001.

Similarly, the agomir‐185‐5p and antagomir‐185‐5p were injected into mouse skin wounds. The results revealed that the wounds in the agomir‐185‐5p group showed the earliest signs of approaching healing at 14 days (Figure 10a,b). Furthermore, the subcutaneous regenerated thickness in the agomir‐185‐5p and EXO groups was thinner than that in the antagomir‐185‐5p group and contained more hair follicle structures (Figure 10c). The COL‐I content in the agomir‐185‐5p group decreased significantly, with the COL‐III/COL‐I ratio approaching 6, while the addition of antagomir‐185‐5p reversed the increase in COL‐I (Figure 10d,e). At the same time, the expression of RhoA and YAP also decreased significantly in the agomir‐185‐5p group (Figure 10f; Figure S9a,b), further confirming the results of in vivo experiments that the miR‐185‐5p‐RhoA/YAP axis targets and regulates COL‐I and COL‐III, thereby promoting scarless wound healing. In summary, functional exosomes obtained through the development of a 3D‐HO culture system can promote scarless wound healing by targeting the miR‐185‐5p‐RhoA/YAP axis to regulate the collagen III/I ratio.

## 4 Discussion

The results of this study demonstrate that 3D‐HO culture significantly increases the yield and functional quality of exosomes derived from human umbilical cord mesenchymal stem cells (hUC‐MSCs). In recent years, research on exosome secretion has focused mainly on 3D culture systems, external mechanical stimulation, and the systematic optimization of culture conditions. These strategies regulate cell secretory behavior at the structural, mechanical, and metabolic levels [36, 37, 38, 39]; however, explorations based on a single model cannot account for the complex changes in the in vivo environment. Therefore, to better simulate physiological growth conditions, we have for the first time proposed and established a 3D‐HO co‐culture strategy, which simultaneously increases the secretion efficiency of exosomes and the healing effect for acute wounds. Compared with traditional culture conditions, we found that the 3D‐HO microenvironment better simulates the physiological interactions between cells and between cells and the ECM, thereby reshaping cell metabolic, polarity and secretory programs. Under such conditions, the cellular activity of hUC‐MSCs increased, stem cell characteristics were maintained, ageing was delayed, and exosome production was significantly increased in each cell line. Moreover, the expression of classical exosome markers and the ESCRT‐related protein CHMP6 was also increased. In addition, experiments in a mouse wound model confirmed the functional effect of exosomes from the 3D‐HO coculture model in promoting the scarless healing of acute wounds, as evidenced by the number of skin appendages observed after skin regeneration and the total amount of collagen regenerated. In this study, the focus was on exploring the precise regulatory effect of exosomes on collagen subtypes. Exosomes can enhance the energy metabolism of cells and thereby increase the overall cell activity. However, through cell low‐energy inhibition experiments, we found that the changes in collagen subtypes were synchronous under conditions of transient low ATP and high ATP; that is, COL‐I and COL‐III increased or decreased simultaneously, with no change in proportion. A change in the COL‐I/COL‐III ratio was observed only in the cells in the single 3D‐HO exosome group, which indicates the specificity of the regulation of the proportion of collagen subtypes by exosomes.

Additionally, miRNA profiling revealed pronounced enrichment of miR‐185‐5p in 3D hypoxic exosomes, indicating that this culture strategy not only amplifies exosome secretion but also promotes the loading of functionally relevant molecules, thereby providing a robust technical basis for therapeutic exosome production in skin repair. Previous studies have shown that miR‐185 (/miR‐185‐5p) inhibits the development of digestive tract tumors [40] and reduces myocardial [41], renal [42], and liver [43] fibrosis. Although the direction and magnitude of the effects of miR‐185‐5p vary across organ fibrosis contexts, likely depending on cell type, disease stage, and the microenvironment, its recurrent appearance within fibrosis/ECM regulatory networks is well recognized. This study revealed a novel role for miR‐185‐5p in controlling collagen subtype proportion and reconstructing the ECM. The results of treating fibroblasts and mouse wound tissues with the miR‐185‐5p mimic and agomir‐185‐5p both confirmed the positive role of miR‐185‐5p in collagen regulation. However, in vivo experiments revealed that antagomir‐185‐5p also exhibited a weak effect on the process of wound healing. This might be because exosomes themselves carry various active components, such as miRNAs, proteins and lipids [44]. These findings indicate that miR‐185‐5p plays a key regulatory role in wound healing but that other trace active factors from exosomes are also involved. These results also reinforce the key role of miR‐185‐5p in the proper remodeling of the collagen ratio and suggest that in the future, the core active components of 3H‐EXOs can be further analyzed to identify synergies among multiple miRNAs or hierarchies of key miRNA combinations.

Among the numerous mechanisms that influence collagen regeneration, we found that RhoA/YAP is regulated by miR‐185‐5p and participates in the regulation of collagen subtypes. Previous studies have shown that the RhoA–YAP axis is the central mechanical transduction module that drives fibrosis [45]. This promotes *α*‐SMA binding, stabilizes the myofibroblast phenotype, and ultimately enhances matrix deposition. Subsequently, YAP evades inhibitory phosphorylation, thereby converting the transient repair response into progressive matrix remodeling and scar maturation [46]. In this study, we quantitatively analyzed the factors related to exosome processing in the RhoA/YAP pathway in fibroblasts and proposed a significant role for this pathway in wound healing. Further inhibition experiments indicated that blocking RhoA/YAP activity had a positive effect on the collagen subtype ratio. In addition, we revealed that miR‐185‐5p can bind to the 3’UTR of RhoA, thereby targeting and inhibiting the expression of RhoA and its downstream inhibitory effect on YAP. Moreover, the results of fibroblast treatment with a YAP activator verified the key role of YAP in the balance of collagen subtypes. Therefore, this study revealed that resetting the mechanical transduction axis (RhoA/YAP) through miR‐185‐5p altered the ECM composition during wound repair.

Although substantial evidence supports the efficacy of 3H‐EXO therapy in promoting scarless healing, the precise nature of the relationships between specific miRNAs and collagen remains to be elucidated. The mass production of exosomes within a 1 L system was achieved in this study. However, further investigation and optimization of the culture process are needed to increase exosome secretion and ensure the consistency of exosome yield and function across different batches. Additionally, there are various purification techniques and industrial production methods for exosomes, which may impact the consistency of exosome quality and their suitability for clinical applications. Considering these challenges, future research should focus on the following topics: (1) The relationships between miRNAs and their various collagen targets should be determined. (2) The exosome purification process should be tailored to the stimulation conditions used to minimize losses at different stages. Previous studies have shown that the surfaces of 3D‐stimulated exosomes contain more integrin proteins. Whether characteristic markers can be specifically targeted to improve the purity and quantity of exosomes is a topic worth exploring in depth.

**FIGURE 11:**
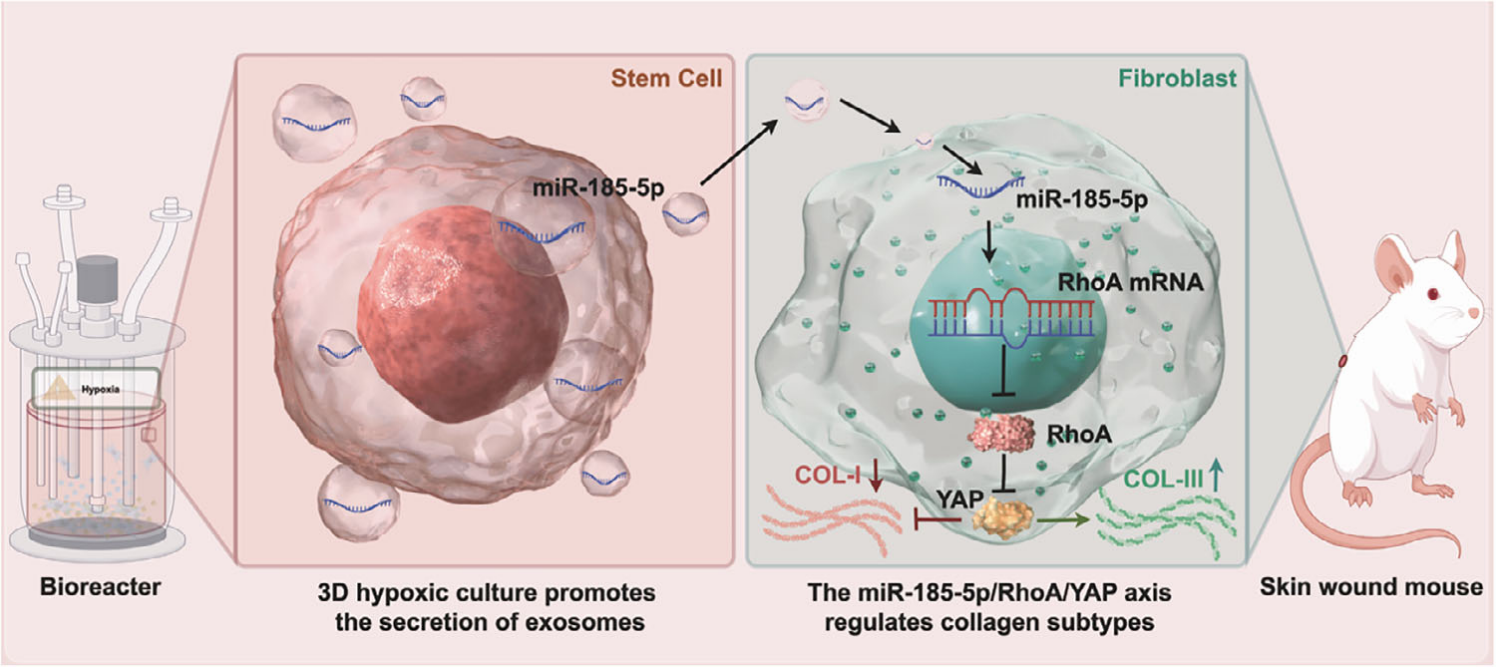
Exosomal miR‐185‐5p targets RhoA to suppress YAP signaling and shift collagen subtype composition. The schematic diagram of 3D hypoxic culture–derived exosomal miR‐185‐5p regulates COL‐I/COL‐III via the RhoA/YAP axis.

In this study, a 3D‐HO suspension culture system for cultivating umbilical cord mesenchymal stem cells to secrete functional exosomes was established. Notably, exosomes generated under these conditions demonstrated significant efficacy in acute wound repair by targeting the RhoA/YAP signaling axis in fibroblasts to regulate the regenerative ratio of COL‐III/COL‐I, thereby preventing hypertrophic scar formation at its source. Within these exosomes, miR‐185‐5p emerged as a primary key regulatory molecule and was highly enriched in 3D‐HO exosomes. Thus, this study presents a pioneering industrial‐scale stem cell culture process that mimics the in vivo microenvironment, yielding a novel biomaterial that promotes scar‐free skin healing (Figure 11).

## Author Contributions

Lihua Yang: conceptualization, investigation, visualization, writing – original draft, writing – review and editing. Yiqun Sun: methodology, writing – original draft, writing – review and editing. Qinbiao Yan: investigation. Ali Mohsin: project administration, writing – review and editing. Kamran Ashraf: investigation. Senyi Gong: visualization. Weifeng Li: investigation. Touseef Ur Rehman: investigation. Yuwen Hu: methodology. Jinzhao He: visualization. Yu Liu: methodology. Meijun Ding: visualization. Lin Qi: writing – review and editing. **Meijin Guo**: conceptualization, funding acquisition, project administration, supervision, writing – review and editing. Ke Xue: conceptualization, funding acquisition, project administration, supervision, writing – review and editing.

## Funding

This work was supported by the National Natural Science Foundation of China (82272290), Hainan Province National Science Foundation of China (822CXTD537, 823MS157), Strategic Priority Research Program of the Chinese Academy of Sciences (XDA16030702).

## Conflicts of Interest

The authors declare no conflicts of interest.

## Supporting information




**Supporting File 1**: advs74187‐sup‐0001‐SuppMat.docx.

**Supporting File 2**: advs74187‐sup‐0002‐TableS1.xlsx.

**Supporting File 3**: advs74187‐sup‐0003‐TableS2.xlsx.

## Data Availability

The data that support the findings of this study are available from the corresponding author upon reasonable request.

## References

[1] S. Meng , Q. Wei , S. Chen , et al., “MiR‐141‐3p‐Functionalized Exosomes Loaded in Dissolvable Microneedle Arrays for Hypertrophic Scar Treatment,” Small 20 (2023): e2305374–e2305374, 10.1002/smll.202305374. 37724002

[2] I. Raote , A.‐H. Rosendahl , H.‐M. Häkkinen , et al., “TANGO1 Inhibitors Reduce Collagen Secretion and Limit Tissue Scarring,” Nature Communications 15 (2024): 3302, 10.1038/s41467-024-47004-1. PMC11043333 38658535

[3] A. Lescoat , J. Varga , M. Matucci‐Cerinic , and D. Khanna , “New Promising Drugs for the Treatment of Systemic Sclerosis: Pathogenic Considerations, Enhanced Classifications, and Personalized Medicine,” Expert Opinion on Investigational Drugs 30 (2021): 635–652, 10.1080/13543784.2021.1923693. 33909517 PMC8292968

[4] R. Ogawa , “Keloid and Hypertrophic Scars are the Result of Chronic Inflammation in the Reticular Dermis,” International Journal of Molecular Sciences 18 (2017): 606, 10.3390/ijms18030606. 28287424 PMC5372622

[5] Y. Guan , H. Niu , Z. Liu , et al., “Sustained Oxygenation Accelerates Diabetic Wound Healing by Promoting Epithelialization and Angiogenesis and Decreasing Inflammation,” Science Advances 7 (2021): 35, 10.1126/sciadv.abj0153. PMC8397271 34452918

[6] P. Shiekh , A. Singh , and A. Kumar , “Exosome Laden Oxygen Releasing Antioxidant and Antibacterial Cryogel Wound Dressing OxOBand Alleviate Diabetic and Infectious Wound Healing,” Biomaterials 249 (2020): 120020, 10.1016/j.biomaterials.2020.120020. 32305816

[7] S. A. Eming , P. Martin , and M. T. Canic , “Wound Repair and Regeneration: Mechanisms, Signaling, and Translation,” Science Translational Medicine 6 (2014), 10.1126/scitranslmed.3009337. PMC4973620 25473038

[8] G. C. Gurtner , S. Werner , Y. Barrandon , and M. T. Longaker , “Wound Repair and Regeneration,” Nature 453 (2008): 314–321, 10.1038/nature07039. 18480812

[9] R. J. Akhurst and A. Hata , “Targeting the TGFβ Signaling Pathway in Disease,” Nature Reviews Drug Discovery 11 (2012): 790–811, 10.1038/nrd3810. 23000686 PMC3520610

[10] F. S. Younesi , A. E. Miller , T. H. Barker , F. M. V. Rossi , and B. Hinz , “Fibroblast and Myofibroblast Activation in Normal Tissue Repair and Fibrosis,” Nature Reviews Molecular Cell Biology 25, no. 8 (2024): 617–638, 10.1038/s41580-024-00716-0. 38589640

[11] Y. Liu , T. Lu , C. Zhang , et al., “Activation of YAP Attenuates Hepatic Damage and Fibrosis in Liver Ischemia‐Reperfusion Injury,” Journal of Hepatology 71, no. 4 (2019): 719–730, 10.1016/j.jhep.2019.05.029. 31201834 PMC6773499

[12] S. Moon , S. Lee , J. A. Caesar , et al., “A CTGF‐YAP Regulatory Pathway is Essential for Angiogenesis and Barriergenesis in the Retina,” Iscience 23, no. 6 (2020): 101184, 10.1016/j.isci.2020.101184. 32502964 PMC7270711

[13] X. V. Dey and K. L. Guan , “Targeting the Hippo Pathway in Cancer,” Nature Reviews Drug Discovery 24 (2020): 852–869, 10.1038/s41573-025-01234-0. 40588515

[14] H. Yu , J. Zhang , X. Liu , and Y. Li , “microRNA‐136‐5p From Bone Marrow Mesenchymal Stem Cell‐Derived Exosomes Facilitates Fracture Healing by Targeting LRP4 to Activate the Wnt/β‐Catenin Pathway,” Bone and Joint Research 10 (2021): 744–758, 10.1302/2046-3758.1012.BJR-2020-0275.R2. 34847690 PMC8712601

[15] J. Behera , A. Kumar , M. J. Voor , and N. Tyagi , “Exosomal lncRNA‐H19 Promotes Osteogenesis and Angiogenesis Through Mediating Angpt1/Tie_2_‐NO Signaling in CBS‐Heterozygous Mice,” Theranostics 11 (2021): 7715–7734, 10.7150/thno.58410. 34335960 PMC8315071

[16] K. Jiang , T. Jiang , Y. Chen , and X. Mao , “Mesenchymal Stem Cell‐Derived Exosomes Modulate Chondrocyte Glutamine Metabolism to Alleviate Osteoarthritis Progression,” Mediators of Inflammation 2021 (2021): 2979124, 10.1155/2021/2979124. 34992497 PMC8724850

[17] A. Matamoros‐Angles , E. Karadjuzovic , B. Mohammadi , et al., “Efficient Enzyme‐Free Isolation of Brain‐Derived Extracellular Vesicles,” Journal of Extracellular Vesicles 13 (2024), 10.1002/jev2.70011. PMC11541858 39508423

[18] V. Suzy and J. M. Lorenowicz , “Mesenchymal Stromal/Stem Cell‐Derived Extracellular Vesicles in Tissue Repair: Challenges and Opportunities,” Theranostics 10 (2020): 5979–5997, 10.7150/thno.40122. 32483432 PMC7254996

[19] M. Proestaki , A. Ogren , B. Burkel , and J. Notbohm , “Modulus of Fibrous Collagen at the Length Scale of a Cell,” Experimental Mechanics 59 (2019): 1323–1334, 10.1007/s11340-018-00453-4. 31680700 PMC6824437

[20] K. Hotary , E. Allen , A. Punturieri , I. Yana , and S. J. Weiss , “Regulation of Cell Invasion and Morphogenesis in a Three‐Dimensional Type I Collagen Matrix by Membrane‐Type Matrix Metalloproteinases 1, 2, and 3,” Journal of Cell Biology 149 (2000): 1309–1323, 10.1083/jcb.149.6.1309. 10851027 PMC2175112

[21] D. Jiang , R. Guo , H. G. Machens , and Y. Rinkevich , “Diversity of Fibroblasts and Their Roles in Wound Healing,” Cold Spring Harbor Perspectives in Biology 15 (2023): a041222, 10.1101/cshperspect.a041222. 36167647 PMC9979851

[22] S. Aashrith , I. Dhiraj , and C. Ovijit , “Cell–Extracellular Matrix Mechanotransduction in 3D,” Nature reviews molecular cell biology 24 (2023): 495–516, 10.1038/s41580-023-00583-1. 36849594 PMC10656994

[23] D. Indana , P. Agarwal , N. Bhutani , and O. Chaudhuri , “Viscoelasticity and Adhesion Signaling in Biomaterials Control Human Pluripotent Stem Cell Morphogenesis in 3D Culture,” Advanced Materials 33 (2021): 2101966, 10.1002/adma.202101966. 34499389

[24] N. Gjorevski , M. Nikolaev , T. E. Brown , et al., “Tissue Geometry Drives Deterministic Organoid Patterning,” Science 375 (2022): aaw9021, 10.1126/science.aaw9021. PMC9131435 34990240

[25] A. Isomursu , K.‐Y. Park , J. Hou , et al., “Directed Cell Migration Towards Softer Environments,” Nature Materials 21 (2022): 1081–1090, 10.1038/s41563-022-01294-2. 35817964 PMC10712428

[26] S. Xu , L. Zhao , Y. Li , et al., “Activating the Healing Process: Three‐Dimensional Culture of Stem Cells in Matrigel for Tissue Repair,” BMC Biotechnology 24, no. 1 (2024): 36–36, 10.1186/s12896-024-00862-5. 38796454 PMC11128131

[27] S. Zhang , K. Wong , M. Wang , et al., “Optimising Administration of MSC Exosomes for Cartilage Repair in the Clinic,” Cytotherapy 22 (2020): S55, 10.1016/j.jcyt.2020.03.077.

[28] N. Bister , C. Pistono , B. Huremagic , J. Jolkkonen , R. Giugno , and T. Malm , “Hypoxia and Extracellular Vesicles: A Review on Methods, Vesicular Cargo and Functions,” Journal of Extracellular Vesicles 10 (2020): 12002, 10.1002/jev2.12002. PMC7710128 33304471

[29] L. Harris , “Hypoxia—A Key Regulatory Factor in Tumour Growth,” Nature Reviews Cancer 2 (2002): 38–47, 10.1038/nrc704. 11902584

[30] L.‐P. Zhu , T. Tian , J.‐Y. Wang , et al., “Hypoxia‐Elicited Mesenchymal Stem Cell‐Derived Exosomes Facilitates Cardiac Repair Through miR‐125b‐Mediated Prevention of Cell Death in Myocardial Infarction,” Theranostics 8 (2018): 6163, 10.7150/thno.28021. 30613290 PMC6299684

[31] B. Oosthuyse , L. Moons , E. Storkebaum , et al., “Deletion of the Hypoxia‐Response Element in the Vascular Endothelial Growth Factor Promoter Causes Motor Neuron Degeneration,” Nature Genetics 28 (2001): 131, 10.1038/88842. 11381259

[32] L. Wang , J. Huang , R. Zhang , et al., “Cullin 5 Aggravates Hypoxic Pulmonary Hypertension by Activating TRAF6/NF‐κB/HIF‐1α/VEGF,” Iscience 26 (2023): 108199, 10.1016/j.isci.2023.108199. 37965157 PMC10641258

[33] Z. Luo , M. Tian , G. Yang , et al., “Hypoxia Signaling in Human Health and Diseases: Implications and Prospects for Therapeutics,” Signal Transduction and Targeted Therapy 7 (2022): 218, 10.1038/s41392-022-01080-1. 35798726 PMC9261907

[34] Y. Wang , Y. Zhang , T. Li , et al., “Adipose Mesenchymal Stem Cell Derived Exosomes Promote Keratinocytes and Fibroblasts Embedded in Collagen/Platelet‐Rich Plasma Scaffold and Accelerate Wound Healing,” Advanced Materials 35 (2023): 2303642, 10.1002/adma.202303642. 37342075

[35] O. Skalli , P. Ropraz , A. Trzeciak , G. Benzonana , D. Gillessen , and G. Gabbiani , “A Monoclonal Antibody Against Alpha‐Smooth Muscle Actin: A New Probe for Smooth Muscle Differentiation,” Journal of cell biology 103 (1986): 2787–2796, 10.1083/jcb.103.6.2787. 3539945 PMC2114627

[36] C. to , “Minimal Information for Studies of Extracellular Vesicles (MISEV2023): From Basic to Advanced Approaches,” Journal of extracellular vesicles 13 (2024): 12451. 10.1002/jev2.12404 PMC10850029 38326288

[37] V. Marina , R. Maja , and S. Harald , “The Many Functions of ESCRTs,” Nature Reviews Molecular Cell Biology 21 (2020): 25–42, 10.1038/s41580-019-0177-4. 31705132

[38] M. Babst , D. J. Katzmann , E. J. Estepa‐Sabal , T. Meerloo , and S. D. Emr , “Escrt‐III,” Developmental Cell 3 (2002): 271–282, 10.1016/S1534-5807(02)00220-4. 12194857

[39] J. Fan , J. Pan , X. Zhang , et al., “A Peptide Derived From the N‐Terminus of Charged Multivesicular Body Protein 6 (CHMP6) Promotes the Secretion of Gene Editing Proteins via Small Extracellular Vesicle Production,” Bioengineered 13 (2022): 4702–4716, 10.1080/21655979.2022.2030571. 35188876 PMC8973635

[40] E. Babaeenezhad , F. Naghibalhossaini , M. Rajabibazl , et al., “The Roles of microRNA miR‐185 in Digestive Tract Cancers,” Non‐Coding RNA 8, no. 5 (2022): 67, 10.3390/ncrna8050067. 36287119 PMC9609348

[41] R. Lin , L. Rahtu‐Korpela , Z. Szabo , et al., “MiR‐185‐5p Regulates the Development of Myocardial Fibrosis,” Journal of Molecular and Cellular Cardiology 165 (2022): 130–140, 10.1016/j.yjmcc.2021.12.011. 34973276

[42] Q. Yuan , T. Xu , Y. Chen , et al., “miR‐185‐5p Ameliorates Endoplasmic Reticulum Stress and Renal Fibrosis by Downregulation of ATF6,” Laboratory Investigation 100, no. 11 (2020): 1436–1446, 10.1038/s41374-020-0447-y. 32514126

[43] B.‐B. Li , D.‐L. Li , C. Chen , et al., “Potentials of the Elevated Circulating miR‐185 Level as a Biomarker for Early Diagnosis of HBV‐Related Liver Fibrosis,” Scientific Reports 6 (2016): 34157, 10.1038/srep34157. 27677421 PMC5039723

[44] R. Kalluri and V. S. LeBleu , “The Biology, Function, and Biomedical Applications of Exosomes,” Science 367 (2020), 10.1126/science.aau6977. PMC7717626 32029601

[45] X.‐H. Zhao , C. Laschinger , P. Arora , K. Szaszi , A. Kapus , and C. A. McCulloch , “Force Activates Smooth Muscle *α*‐Actin Promoter Activity Through the Rho Signaling Pathway,” Journal of Cell Science 120, no. 10 (2007): 1801–1809, 10.1242/jcs.001586. 17456553

[46] F. Liu , D. Lagares , K. M. Choi , et al., “Mechanosignaling Through YAP and TAZ Drives Fibroblast Activation and Fibrosis,” American Journal of Physiology‐Lung Cellular and Molecular Physiology 308, no. 4 (2015): L344–L357, 10.1152/ajplung.00300.2014. 25502501 PMC4329470

