# Supplementary material for: miR‐185‐5p Derived From hUC‐MSC Exosomes via Suspension Culture Under Hypoxic Conditions Promotes Scarless Wound Healing in Mice by Precisely Regulating Collagen I/III Regeneration
Source: Adv Sci (Weinh). 2026 Feb 19;13(17):e16120. doi: 10.1002/advs.202516120 (PMC13042918; doi:10.1002/advs.202516120)
Supplement: Supplementary file 1 — Supporting File 1: advs74187‐sup‐0001‐SuppMat.docx. [file ADVS-13-e16120-s003.docx]

**Supplementary** **figtures**

**Methods：**

**1. Characterisation of cell culture processes**

Energy metabolism and cellular activity during cell culture can be indicative of changes in the growth process.The study examined the cell activity, density, glucose and lactate content of umbilical cord MSCs cultured under different conditions (normoxia, hypoxia) on a daily basis. Cell activity and density were measured using a cell counter, and culture supernates and cell sampling were performed on a sterile operating table. The culture media were then aspirated into a T175 square bottle and transferred into a centrifuge tube (300g, 5 minutes). The resulting pellet was then resuspended in the biochemical analyser for glucose and lactate content. Concurrently, 5 mL of 0.125% trypsin (Gibco) was added to the bottle containing only cells, and the contents were left to digest for a period of five minutes. Thereafter, 10 mL of complete medium was added and the contents were subjected to repeated agitation, after which the mixture was transferred to a centrifuge tube and subjected to centrifugation at 300 g for a period of five minutes. The resultant precipitate was then resuspended in PBS, and subsequently placed into a counter to be counted directly.

**2.** **Cell Activity Assay**

The fundamental prerequisite for cells to produce exosome is to be able to grow normally, and the optimal growth conditions for cells can be screened by the detection of cellular CCK8 activity under different conditions. Following inoculation of cells in 96-well plates, cck8 solution (Biyuntian) was added on days D0, D1, D2 and D3 of growth and co-cultivated for 30 minutes at 37 degrees Celsius. The plates were then placed in an enzyme marker for detection at 450nm.

**3.** **Cell Activity Assay**

Mitochondrial function and lysosomal senescence serve as critical indicators of cellular senescence. The assessment of lysosomal senescence entailed the quantification of β-galactosidase (Senescence β-Galactosidase Staining Kit, Beyotime Biotechnology, China), while the evaluation of mitochondrial potential was facilitated by the use of ROS and mito-tracker red to ascertain mitochondrial function and activity (Mitochondrial Membrane Potential and Apoptosis Detection Kit with Mito-Tracker Red CMXRos and Annexin V-FITC, Beyotime Biotechnology, China).

**4. Animal tissue staining**

The recovery of the wounds was documented at 0, 3, 7, 10, and 14 days during the feeding period. On the 7th and 14th days, samples of the wounds and the surrounding tissues were obtained for Masson staining analysis.

**5.** **Effects of exosome endocytosis on the Fibroblast**

Exosomes were stained with PKH26 at 37 °C for 30 minutes. When the cells in the six-well plate reached 50-70% confluence, the total medium was replaced with fresh medium, and the sterile exosome labeling solution was added to incubate for 1-3 hours. Subsequently, exosome migration and endocytosis were observed under a microscope.

**6. Scratch assay**

The 200 μL tip was used to draw a narrow line on the plate once the cells in the six-well plate reached 50-60% confluence. Subsequently, the plates were incubated for an additional 24 hours to monitor cell growth and migration.

**7. Immunofluorescence analysis of G-actin and ROS levels in fibroblasts**

The functional activity of fibroblasts (Fbs) treated with exosomes, including their ability to G-actin and ROS, was determined. For the ROS analysis, some of the Fbs were stained using a ROS kit (Wanlei Biotech, China) and observed under a fluorescence microscope (MF52-N+MSX2; China). For the G-actin analysis, some of the Fbs were stained using a Cytoskeleton (actin monomer; G-ACTIN) Red fluorescence staining kit (Hepeng Biotech, China) and observed under a confocal laser scanning microscope (FV4000; Olympus, Japan).

**8. Western blots levels in human fibroblasts and mouse skin tissue**

To prepare samples for WB, prechilled lysates were added to Fbs and skin tissue, followed by intermittent sonication for 1.0 minutes and centrifugation in a cryogenic centrifuge at 4 °C for 30 minutes at 500 × g to obtain the proteins in the supernatant. Moreover, the protein concentration was determined using a BCA protein concentration assay kit (Beyotime, China). Afterwards, a Yeasen Fast Gel Kit (China) was used to prepare 10% gels, and the denatured proteins were loaded with 5× loading buffer (Beyotime, China) and separated for 2 hours at 80 V. Protein transfer was performed by attaching the protein gel to a polyvinylidene fluoride (PVDF) membrane for 1.0 hours at 110 V, after which the PVDF membrane was immersed in skim milk for 2 hours at room temperature, followed by incubation with the primary antibody for 12 hours at 4 °C. Finally, the membrane was incubated with secondary antibody for 2 hours at room temperature, after which the protein was visualised using enhanced chemiluminescence (ECL; Yeasen Biotech, China).

**Fig.S1**

**
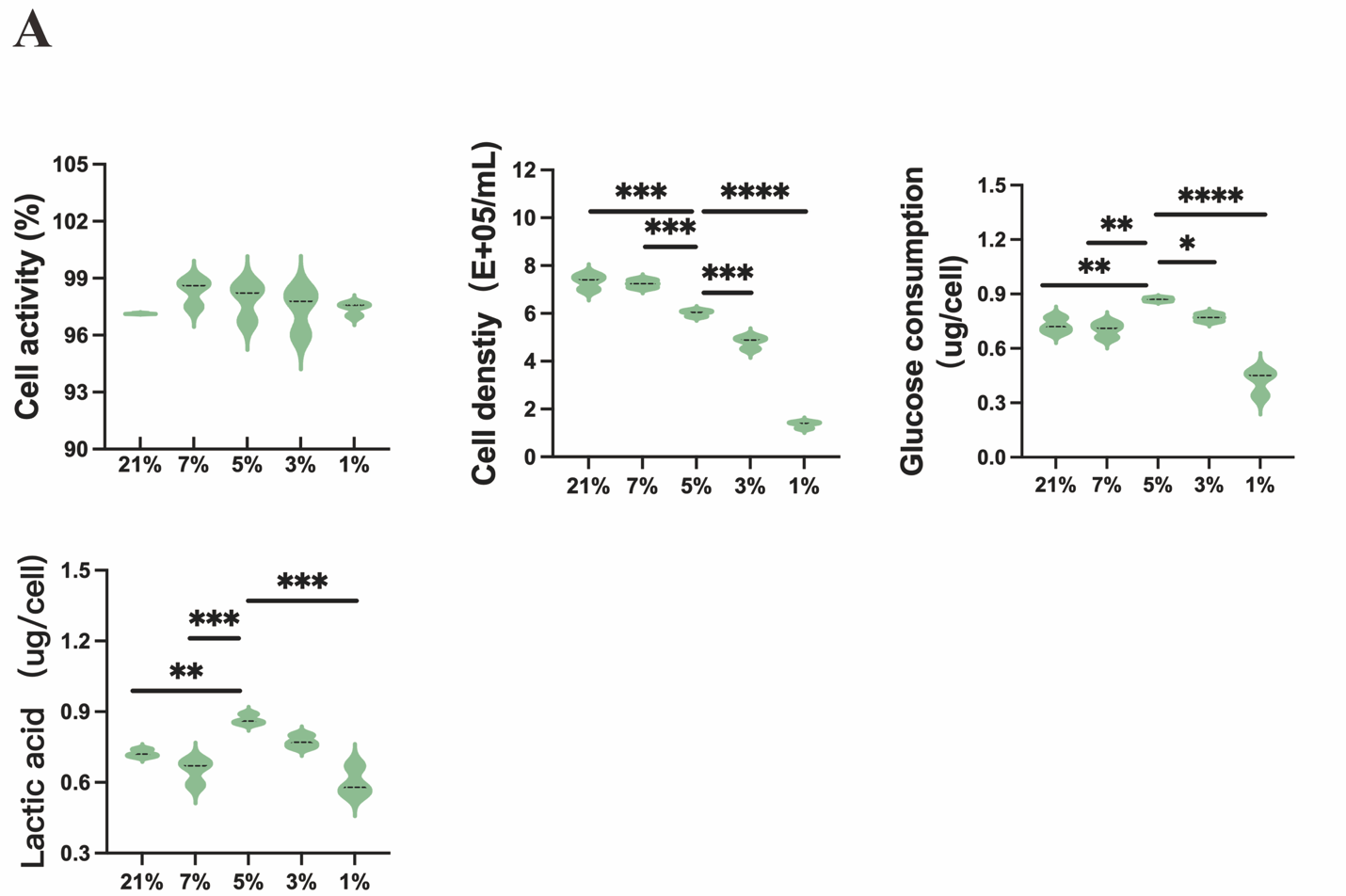
**

**Fig.S1 The physiological functions of stem cells under different Oxygen concentration.** A Two-dimensional cell culture parameters under different oxygen concentrations: cell activity, cell density, glucose consumption, lactic acid. 21% as normoxic, 7%-1% as hypoxic. **p*<0.05, ***p*<0.01, ****p*<0.001, *****p*<0.0001.

**Fig.S2**


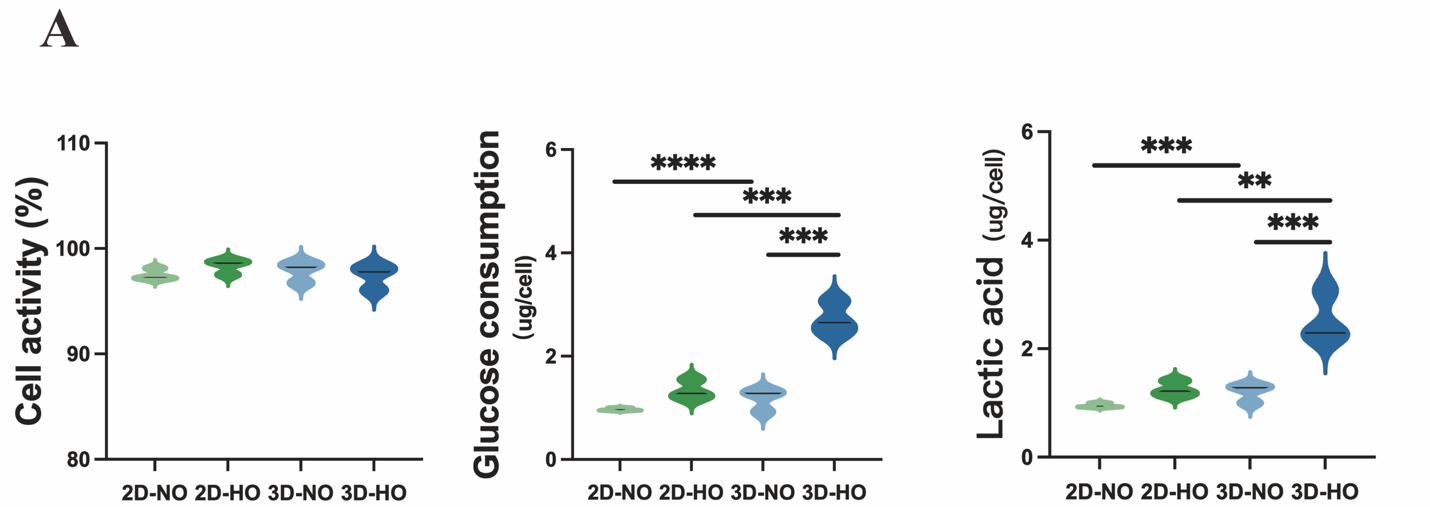


**Fig.S2** **The physiological functions of stem cells under different culture conditions.** A Cell culture parameters under different culture conditions: cell activity, glucose consumption, lactic acid. **p* < 0.05, ***p* < 0.01, ****p* < 0.001, *****p* < 0.0001.

**Fig.S3**


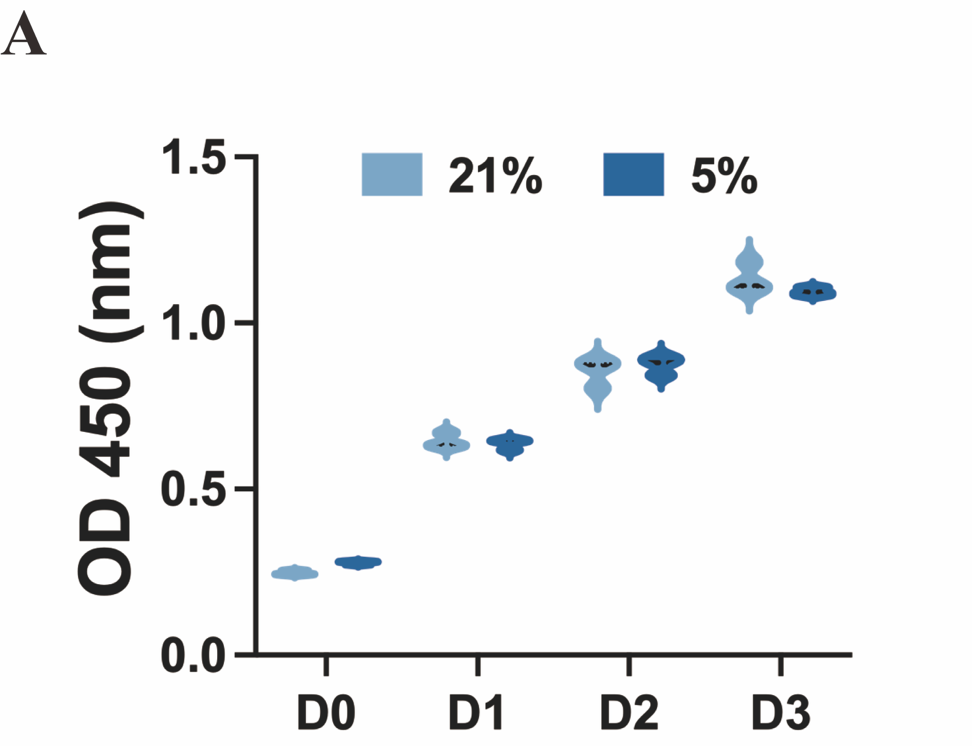


**Fig. S3 The CCK8 values of stem cells under different culture conditions.** A The CCK8 of cells under different culture conditions.

**Fig.S4**

**A**


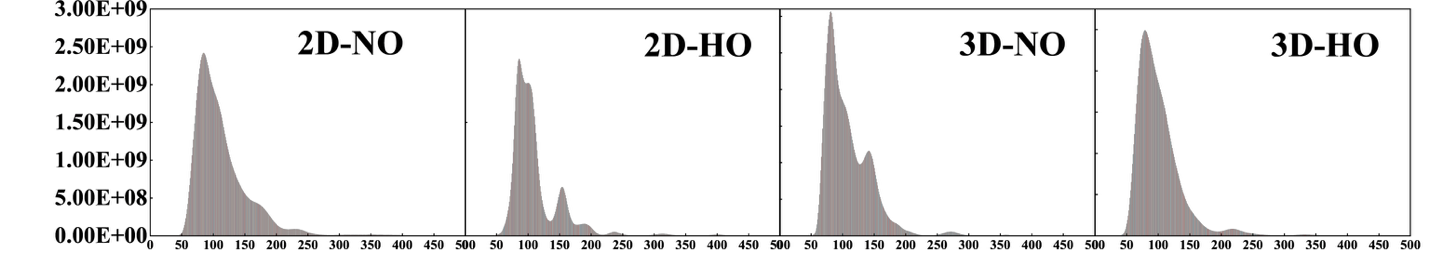


**Fig.S4** **The number of exosome particles under different culture conditions.** A The cell exosome particles under different culture condition.

**Fig.S5**

**A**


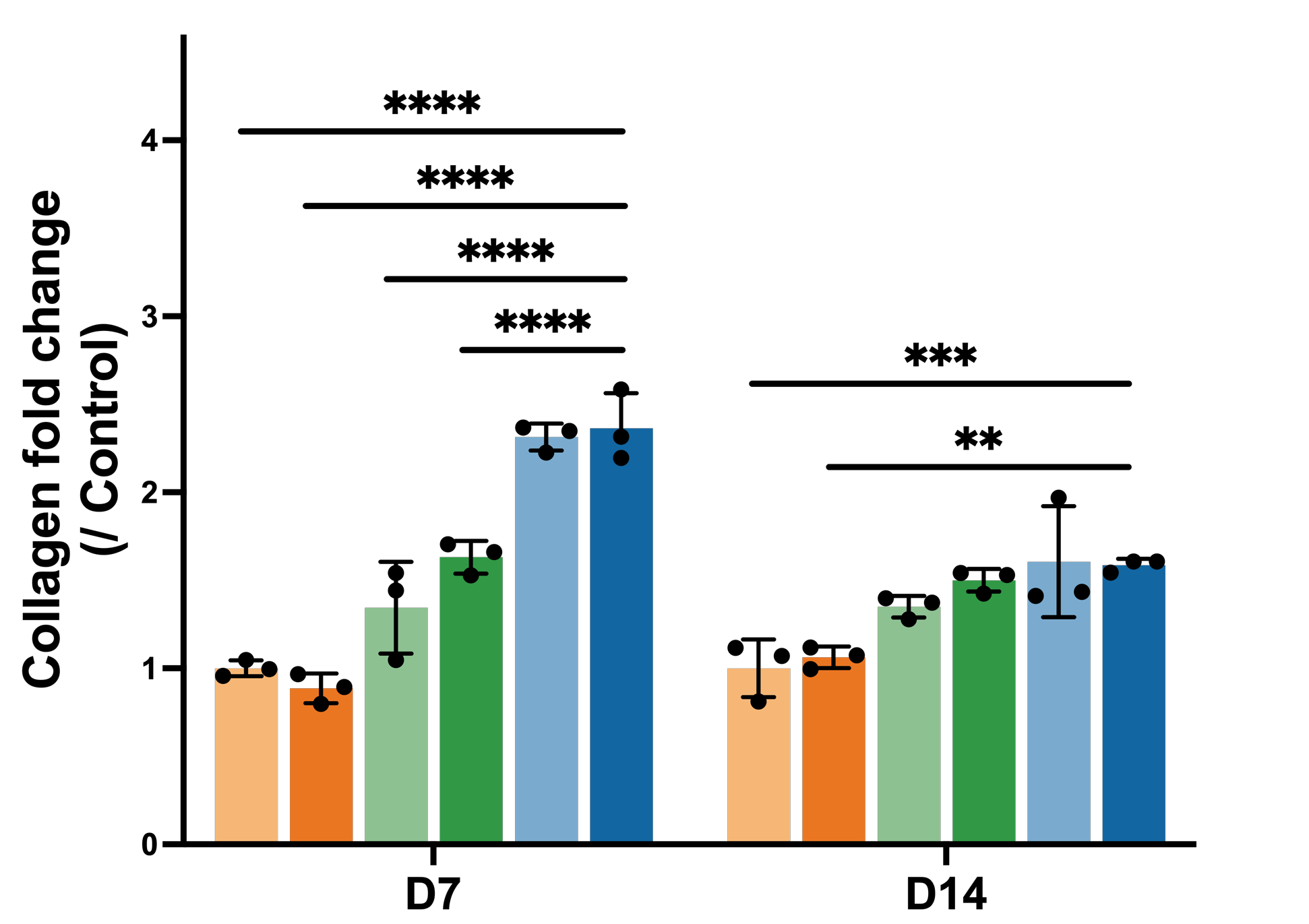


**Fig. S5** **The effect of exosomes on collagen formation during wound healing in mice.** A The collagen fold change in different time points in the 3H-NO condition

**Fig.S6**


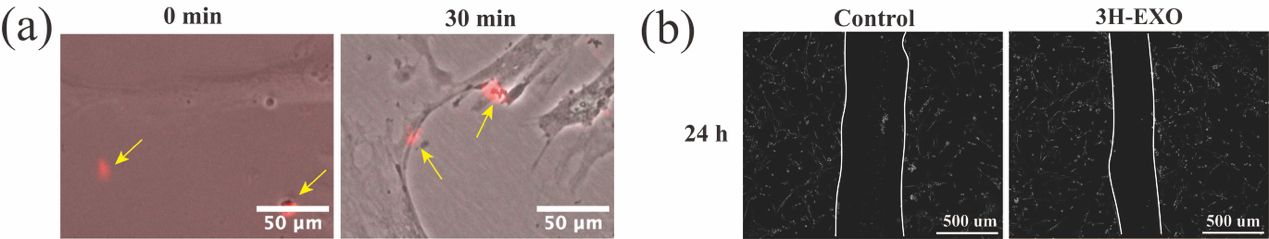


**Fig. S6 The absorption effect of fibroblasts on exosomes** (a) PKH staining labeled exosomes. (b) Migration of fibroblasts after 24 hours of exosome treatment.

**Fig. S7**


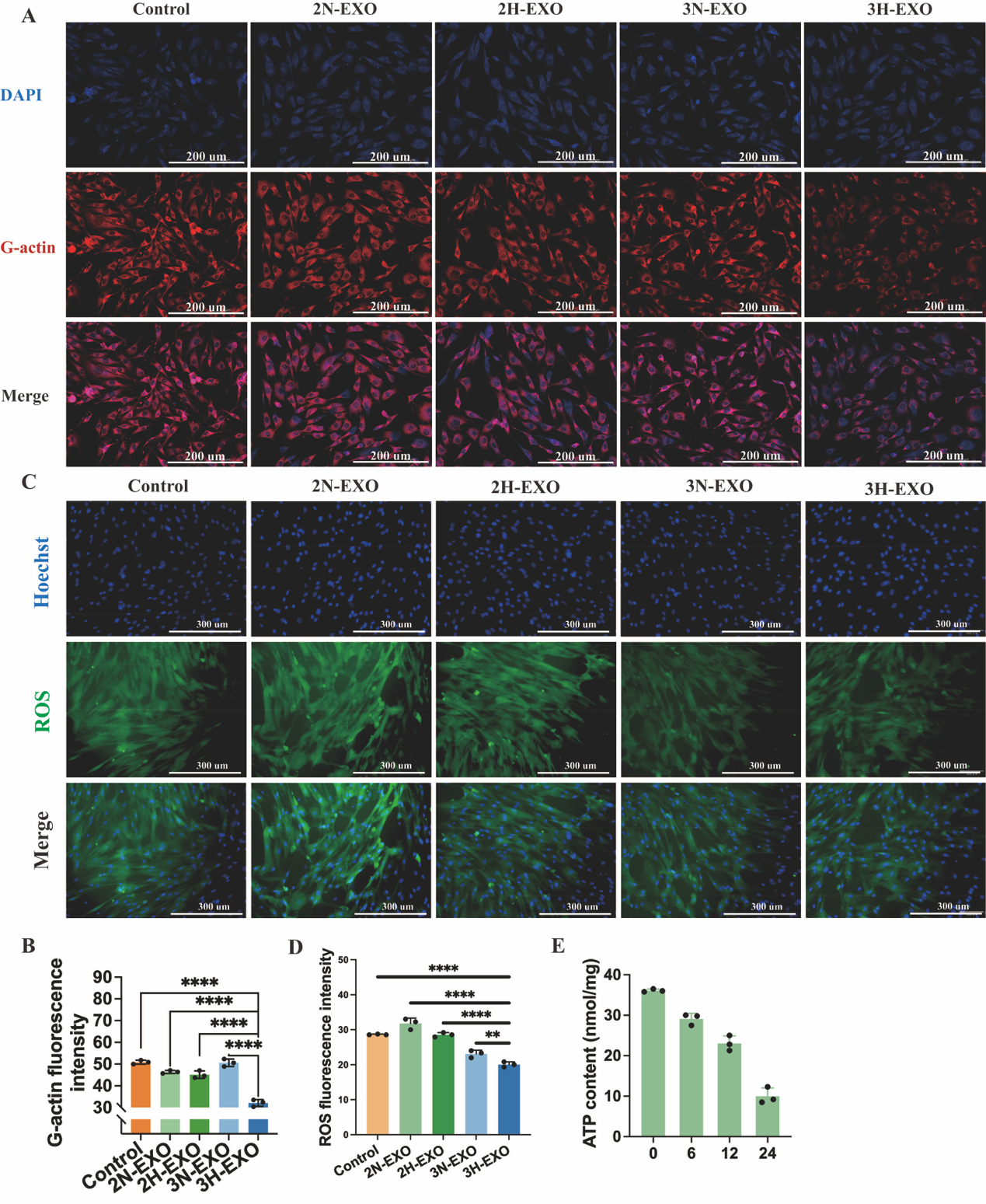


**Fig. S7** **Functional analysis of fibroblasts after exosome treatment.** A The G-actin fluorescence quantification of fibroblasts treated by 3H-EXO (200 um). B The ROS fluorescence quantification of fibroblasts treated by 3H-EXO (300 um). C Statistical Analysis of ROS fluorescence quantification. D Changes in ATP content of fibroblasts at different times in serum-free low-glucose culture.

**Fig.S8**


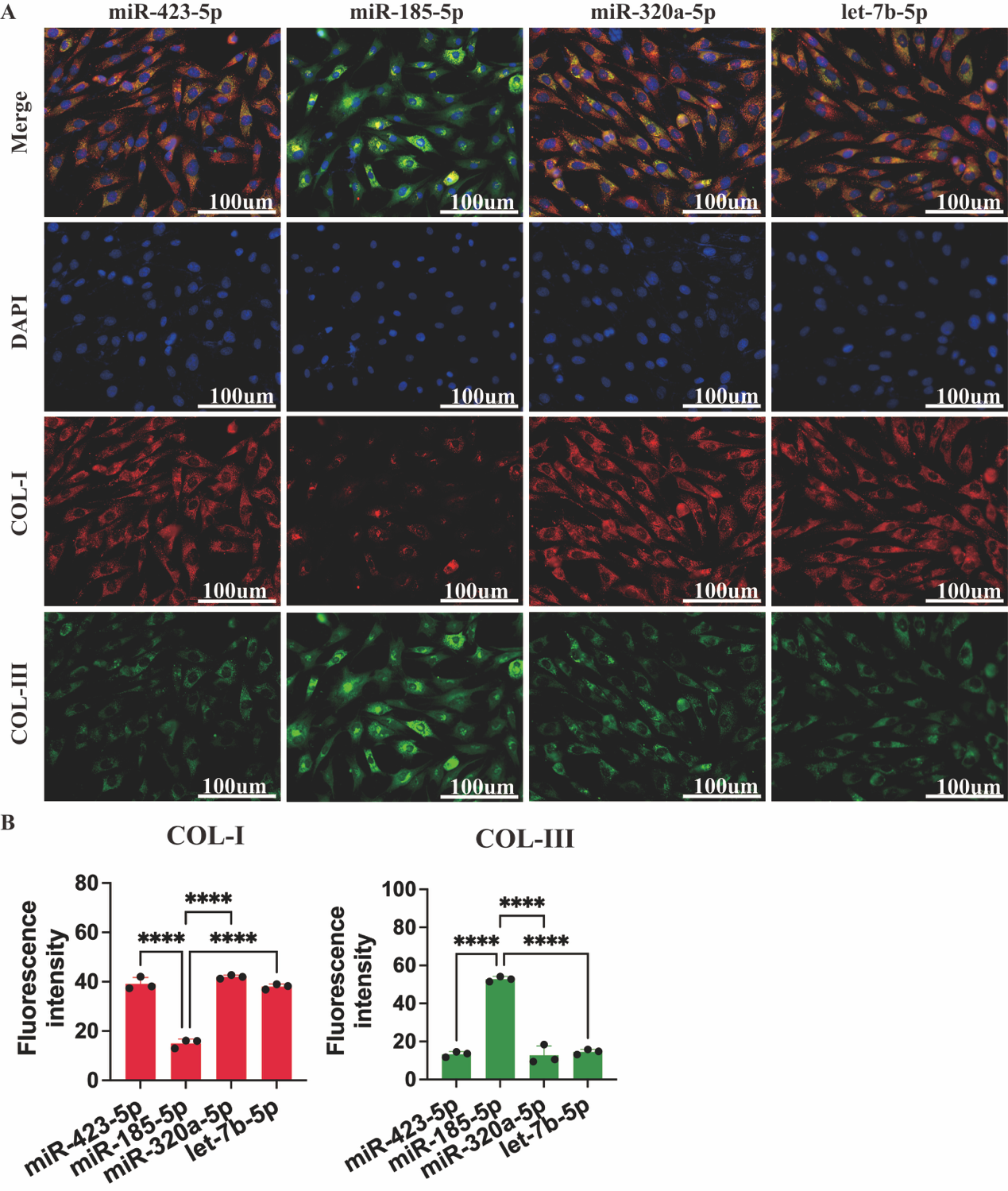


**Fig. S8** **The influence of different miRNAs on collagen formation in fibroblasts.** A The effect of miR-423-5p, miR-320a-5p, miR-185-5p, and let-7b-5p on fibroblasts COL-I and COL-III content. B Immunofluorescence quantitative analysis results of COL-I/COL-III.

**Fig.S9**

**
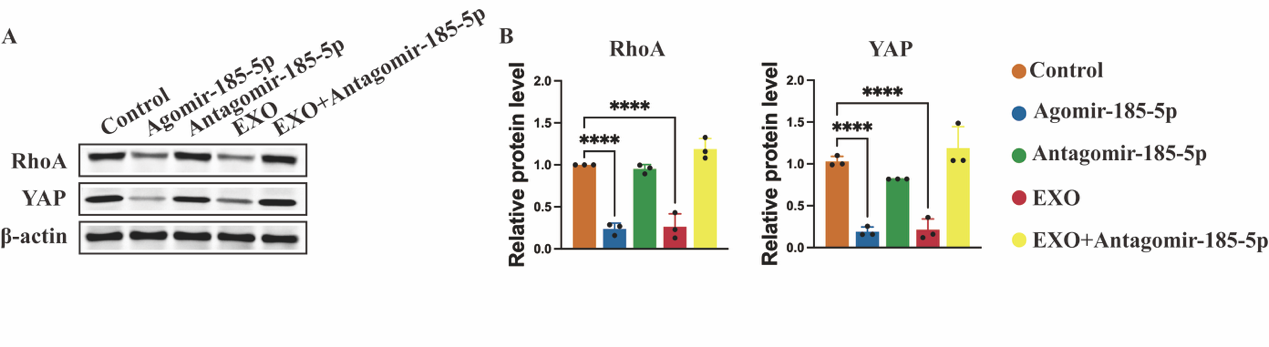
**

**Fig. S9** **Quantitative analysis of western blot in mouse skin tissue after antagomir treatment.** A. The protein levels of RhoA and YAP on mouse skin tissue by antagomir-185-5p. B. Western blot quantitative statistical analysis.
